# Disruption of the plant-specific *CFS*1 gene impairs autophagosome turnover and triggers EDS1-dependent cell death

**DOI:** 10.1038/s41598-017-08577-8

**Published:** 2017-08-17

**Authors:** Arpaporn Sutipatanasomboon, Stefanie Herberth, Ellen G. Alwood, Heidrun Häweker, Britta Müller, Mojgan Shahriari, Anke Y. Zienert, Birger Marin, Silke Robatzek, Gerrit J. K. Praefcke, Kathryn R. Ayscough, Martin Hülskamp, Swen Schellmann

**Affiliations:** 1 Botanik III, Biocenter, Universtiy of Cologne, Zülpicher Str. 47B, 50674 Cologne, Germany; 2 Department of Biomedical Science, The University of Sheffield, Western Bank Sheffield, S10 2TN United Kingdom; 3 The Sainsbury Laboratory, Norwich Research Park, Norwich, NR4 7UH United Kingdom; 4 Institut für Biologie II, University of Freiburg, Schänzlestrasse 1, 79104 Freiburg i. Br., Germany; 5 Institut für Genetik, Universtiy of Cologne, Zülpicher Str. 47A, 50674 Cologne, Germany; 6 Botanik I, Biocenter, Universtiy of Cologne, Zülpicher Str. 47B, 50674 Cologne, Germany; 7 Division of Haematology/Transfusion Medicine, Paul-Ehrlich-Institut, Federal Institute for Vaccines and Biomedicines, Paul-Ehrlich-Str. 51-59, 63225 Langen, Germany

## Abstract

Cell death, autophagy and endosomal sorting contribute to many physiological, developmental and immunological processes in plants. They are mechanistically interconnected and interdependent, but the molecular basis of their mutual regulation has only begun to emerge in plants. Here, we describe the identification and molecular characterization of *CELL DEATH RELATED ENDOSOMAL FYVE*/*SYLF PROTEIN 1* (*CFS1*). The CFS1 protein interacts with the ENDOSOMAL SORTING COMPLEX REQUIRED FOR TRANSPORT I (ESCRT-I) component ELCH (ELC) and is localized at ESCRT-I-positive late endosomes likely through its PI3P and actin binding SH3YL1 Ysc84/Lsb4p Lsb3p plant FYVE (SYLF) domain. Mutant alleles of *cfs1* exhibit auto-immune phenotypes including spontaneous lesions that show characteristics of hypersensitive response (HR). Autoimmunity in *cfs1* is dependent on ENHANCED DISEASE SUSCEPTIBILITY 1 (EDS1)-mediated effector-triggered immunity (ETI) but independent from salicylic acid. Additionally, *cfs1* mutants accumulate the autophagy markers ATG8 and NBR1 independently from EDS1. We hypothesize that CFS1 acts at the intersection of autophagosomes and endosomes and contributes to cellular homeostasis by mediating autophagosome turnover.

## Introduction

Cell death is an important process for plant development and responses to stress^1^. Different types of cell death have been classified based on morphological and biochemical criteria^2, 3^. Vacuolar cell death is characterized by the increase in the size of the central vacuole that engulfs portions of the cytosol and leaves behind empty cell-walls^3^. By contrast, necrosis is a form of an acute cell death that often results from environmental stress. It is characterized by swelling of mitochondria, production of reactive oxygen species, and early rupture of the plasma membrane leading to protoplast shrinkage. It often leaves behind an unprocessed cell corpse^3^. A combination of both types has been observed in the case of pathogen-associated cell death such as hypersensitive response (HR). HR is often accompanied by callose apposition at the cell walls next to the dying cell and organelle swelling^3, 4^.

HR often occurs when the plant immune system is challenged^1^. Plants have evolved two layers of immunity: pathogen-associated molecular pattern (PAMP)-triggered immunity (PTI) and effector-triggered immunity (ETI)^5^. PTI is activated when cell surface-localized receptors recognize PAMPs. ETI is activated when a pathogen unloads virulent effectors with the aim to suppress PTI and invade the cell^5^. These effectors are recognized by intracellular nucleotide-binding leucine-rich repeat receptor proteins (NB-LRRs) or resistance (R)-gene products directly or indirectly by guarding effector-modified host targets^6^.

NB-LRRs are often divided into two classes based on domain organization and the subsequent signal transduction triggered. Signals generated from coiled-coiled (CC)-NB-LRRs are mediated by *NON RACE-SPECIFIC DISEASE RESISTANCE* (*NDR1*); whereas signals generated from Toll-interleukin-1-receptor domain (TIR)-NB-LRRs are mediated by *ENHANCED DISEASE SUSCEPTIBILITY* (*EDS1*)^7^. These signals invoke both local resistance at the infection site and systemic acquired resistance (SAR). SAR protects the whole plant through an accumulation of salicylic acid (SA) and upregulation of defense-related genes such as *PATHOGENESIS-RELATED GENES (PR)* ^8^. Accumulation of SA requires functional *SALICYLIC ACID INDUCTION DEFICIENT* 2 (*SID2*) gene^9^ that is required for SA production in chloroplasts, and *SID1*/*ENHANCED DISEASE SUSCEPTIBILITY* 5 (*EDS5*)^10^ that exports SA to the cytosol^11^. Mutants of genes involved in SAR often spontaneously initiate HR-like lesions^12, 13^. In some cases, they also display a runaway cell death phenotype, where once initiated, cell death cannot be restricted^12, 14^. In addition to SA accumulation and *PR* gene upregulation, *EDS1* is also required for autophagy induction in response to pathogen infection^15^.

Autophagy is a conserved degradation pathway that contributes to cellular homeostasis by degrading unwanted cytosolic components, damaged organelles or invasive pathogens. As described for yeast and animals, autophagy plays an important role in development and stress responses in plants^16^. Several mutants of genes involved in autophagy are sensitive to nutrient-limiting conditions and display early senescence^17–19^. It was also demonstrated in *Picea abies* embryonic suspensor that inhibition of autophagy partially contributes to the switch from vacuolar to necrotic cell death^20^.

Cargoes destined for autophagic degradation are engulfed by a *de novo* formed phosphatidylinositol 3-phosphate (PI3P)- and phosphatidylethanolamine (PE)-rich double membrane structure, the autophagosome^21^. Autophagosome initiation and formation is mediated by autophagy cargo receptors and a group of autophagy-related proteins (ATG). ATG proteins function in a cascade leading to conjugation of ATG8 to PE that expands the double membrane^16, 21^. Mature autophagosomes are delivered to the vacuole, where they are degraded along with cargo receptors and ATG8. Alternatively, autophagosomes can also fuse with multivesicular bodies (MVBs), forming amphisomes before vacuolar degradation^21^.

MVBs are late endosomes that contain transmembrane proteins sorted into the intraluminal vesicles (ILV) and, like autophagosomes, are degraded upon vacuolar fusion. The sorting of transmembrane proteins into ILV requires the function of the ENDOSOMAL SORTING COMPLEX REQUIRED FOR TRANSPORT (ESCRT) that consists of five subcomplexes: ESCRT-0 to –III and the Vacuolar protein sorting (Vps) 4-associated complex. In yeast and animals, ESCRT is initiated by ESCRT-0, which is formed by Vps27p and HseIp^22^. Vps27 is required for the recruitment of Vps23p (named ELC in *Arabidopsis* ^23^), a part of the ESCRT-I complex, to the endosomal membrane to initiate ESCRT sorting^22, 24^. In plants, all members of ESCRT-I to III and Vps4-associated complex exist, but neither Vps27 nor HseI have been identified^25^. Instead, proteins similar to budding yeast (*Saccharomyces cerevisiae*) TARGET OF MYB1 (TOM1)^26^ and FYVE1/ Fab 1, YOTB, Vac 1, and EEA1 (FYVE) DOMAIN PROTEIN REQUIRED FOR ENDOSOMAL SORTING 1 (FREE1) were hypothesized to fulfill ESCRT-0 function in plants^27, 28^.

A number of *Arabidopsis* ESCRT component mutants display deficiency in autophagy^29^. The lack of the ESCRT-III-related CHARGED MULTIVESICULAR BODIES PROTEIN 1 (CHMP1) causes a delay in autophagosome maturation that ultimately results in the accumulation of its cargo proteins^30^. Arabidopsis plants overexpressing the ESCRT-III component, VPS2.1, mutants of VPS2.1 deubiquitinating enzyme DUB, ASSOCIATED MOLECULE WITH THE SH3 DOMAIN OF STAM1 (AMSH1) and mutants of FYVE1/FREE1 show autophagosome accumulation due to decreased vacuolar delivery of autophagosomes^31, 32^.

In this study, we present the molecular and genetic characterization of *Arabidopsis* At3g43230/CFS1. Based on the combination of its FYVE and YSC84 Actin binding (YAB)/SYLF domains, this protein is unique to and conserved within the plant kingdom. We show that CFS1 co-localizes and interacts with the ESCRT-I component, ELC and binds specifically to PI3P. In addition, we also demonstrate that disruption of CFS1 affects autophagy, proteostasis and EDS1-mediated HR. We propose that CFS1 is involved in autophagosome turnover, thereby maintaining cellular homeostasis. In the mutant, disturbed homeostasis activates EDS1-dependent HR.

## Results

### Identification of CFS1

**Figure 1:**
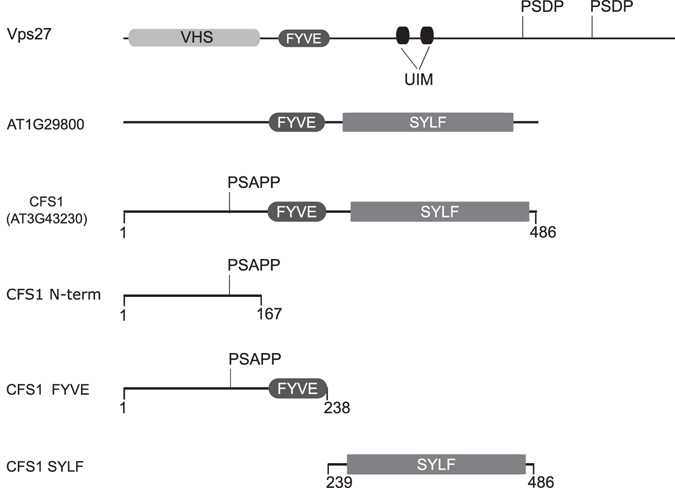
Schematic representation of Vps27p, AT1G29800 and CFS1. The yeast protein Vps27p contains a VPS27, Hrs and STAM (VHS) and FYVE domain, two ubiquitin-interacting motifs (UIM) and two PSDP motifs. AT1G29800 and CFS1 carry a FYVE and a SYLF domain, but only CFS1 contains the conserved PSAPP motif. CFS1 fragments used for localization and membrane interaction studies are shown. Numbers correspond to the amino acid positions.

Plant genomes do not contain obvious ESCRT-0 homologs^25^. To identify possible candidates for ESCRT-0 function in *Arabidopsis* we performed BLAST searches using the full-length sequence of yeast Vacuolar protein sorting 27p (Vps27p). As best matches, we identified two proteins with sequence similarities to the Vps27p FYVE domain, At1g29800 and At3g43230. Both are conserved in the plant kingdom, and homologs can be found in all major groups of land plants and green algae (Supplementary Figure S1). Based on the mutant phenotype caused by *At3g4323*0 loss-of-function, we named it *CELL DEATH RELATED ENDOSOMAL FYVE/SYLF PROTEIN 1* (*CFS1*). CFS1 and At1g29800 share the same domain structure. In addition to the FYVE domain, they carry a DOMAIN OF UNKNOWN FUNCTION 500 (DUF500) at their C-terminus (Fig. 1). This domain has been termed YSC84 Actin binding (YAB) domain in yeast^33^ and SYLF in the mammalian system^34^. The FYVE and the SYLF domains of CFS1 and At1g29800 display a high degree of amino acid sequence conservation with 63% identity (75% similarity) in the FYVE domain and 59% identity (71% similarity) in the SYLF domain. Their N-terminal region by contrast, is less conserved with only 18% identity and 34% sequence similarity. One striking feature in the N-terminal part is the presence of a conserved PSAPP motif in CFS1 that is absent from At1g29800 (Figs 1 and S2A). P(S/T)XP motifs are known from yeast and mammals to mediate binding to the Vps23/TSG101 subunit of ESCRT-I^22, 35^. We found that CFS1 but not At1g29800 interacts with plant VPS23/ELC in a pair-wise yeast-two-hybrid (Y2H) experiment and LUMIER coprecipitation assays (Supplementary Figure S2B,C). We, therefore, focused our investigations on CFS1.

### *cfs1* mutants display a cell death phenotype

To address *CFS1* function, we analyzed three homozygous T-DNA alleles. All three alleles contained single T-DNA insertions located in the first exon of the *CFS1* CDS (Supplementary Figure S3A). In two alleles, no detectable *CFS1* transcripts were found (*cfs1-1*, *cfs1-2*). The third allele showed expression of the C-terminal fragment containing the SYLF domain (*cfs1-3*, Supplementary Figure S3A).

**Figure 2:**
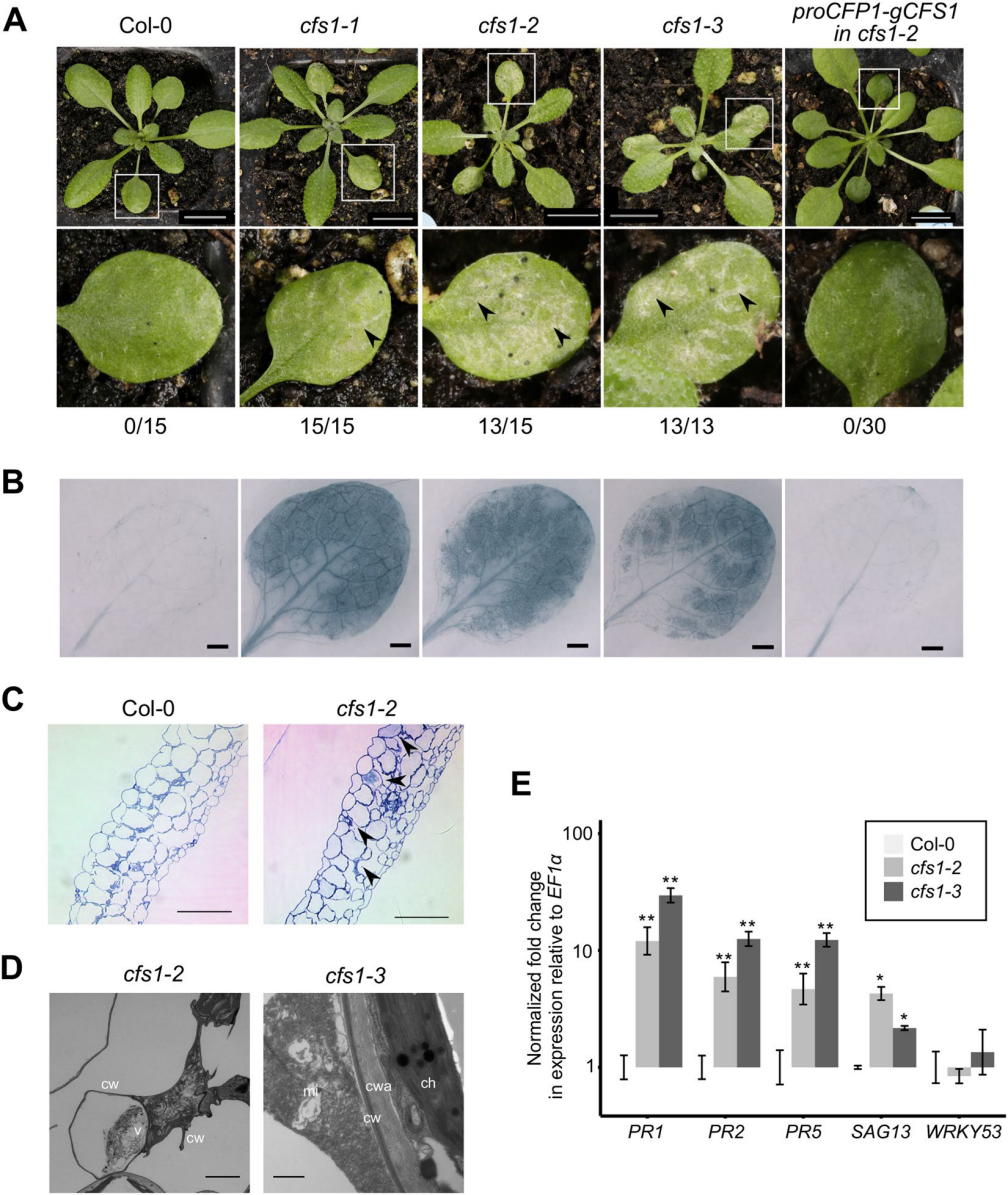
*cfs1* mutants display an autoimmune phenotype. (**A**) Three-week old Col-0, *cfs1-1*, *cfs1-2*, *cfs1-3* and *cfs1-2* expressing the CFS1 genomic fragment driven by its native promoter (*pCFS1-gCFS1* in *cfs1-2*). Squares indicate the digitally magnified region below. Arrowheads indicate lesions. Scale bars: 1 cm. The values below each row represent the number of plants displaying lesions and total number of plants observed from one representative experiment, respectively. (**B**) Trypan blue staining of the first and second leaf of a three-week old plant. The genotype for each image is indicated in the respective panel A. Scale bars: 1 mm. (**C**) Semi-thin cross-sections of Col-0 and *cfs1-2* first leaf of a three-week old plant. Samples were stained with toluidine blue. Arrowheads indicate dead cells. Scale bars: 100 µm. (**D**) Representative TEM pictures of cells in leaf 1 or 2 of three-week old *cfs1* plants. In *cfs1-2*, dead cells contain the unprocessed cell corpse, a characteristic of necrotic cell death (Scale bar is 5 µm). In *cfs1-3*, cell wall appositions between living and the neighboring dead cells, degraded mitochondria and chloroplast are shown (Scale bar is 0.1 µM). (c: cytosol, ch: chloroplast, cw: cell wall, cwa: cell wall appositions, mi: mitochondria, v- vacuole). Dead cells could be readily observed in all *cfs1* alleles but never in any of the Col-0 samples. (**E)** Upregulation of *PR1*, *PR2*, *PR5*, SAG13 and *WRKY53* expression in *cfs1-2* and *cfs1-3* compared with Col-0. Values are plotted in exponential scale (log_10_). Error bars represent the standard error of three biological replicates with two technical replicates. Double asterisks indicate statistical significance at *p* ≤ *0*.01, and an asterisk indicates statistical significant at *p* ≤ 0.05 (ANOVA test, followed by Tukey’s Honest Significance Test).

Most of *cfs1* plants in all three alleles formed spontaneous lesions on leaves 1 and 2 at the 6-leaves stage (Fig. 2A). Lesions started close to the leaf veins and developed to the following leaves, forming large chlorotic areas (Fig. 2A). To test whether they are caused by cell death, we stained wild type (Col-0) and *cfs1* plants with trypan blue (Fig. 2B). We could only sporadically observe stained cells that are indicative of cell death in wild type plants (Col-0) but strong staining in all *cfs1* alleles, in a pattern that mirrored the lesions (Fig. 2B).

This phenotype was rescued by introducing the *CFS1* genomic region (Fig. 2A) demonstrating that the loss of *CFS1* causes cell death.

### Cell death in *cfs1* alleles shows cellular features of hypersensitive response (HR)

To gain insights into the type of cell death, we analyzed cross sections of *cfs1*-2 mutant leaves by light microscopy and transmission electron microscopy (TEM). We observed dead cells in the ground tissue that had shrunk cytoplasm close to the leaf veins that were absent from Col-0 (Fig. 2C). In many cases, the cell wall had retracted from the neighboring cells, leaving vacuole-less remnants with condensed cytoplasm and degraded chloroplasts and mitochondria (Fig. 2D). Cells that are located next to dead cells frequently developed cell wall appositions (Fig. 2D). Together, the cellular patterns observed in *cfs1* are reminiscent of HR-related cell death. Next, we assessed whether HR marker genes were up-regulated using quantitative RT-PCR. In lesion developing *cfs1-2 and cfs1-3*, we observed 5–20fold up-regulation of the *PR1*, *PR2*, *PR5* and *SAG13* genes; while the expression of senescence marker, *WRKY53* remained unchanged from Col-0 (Fig. 2E).

### Cell death in *cfs1* is mediated by EDS1-dependent ETI

**Figure 3:**
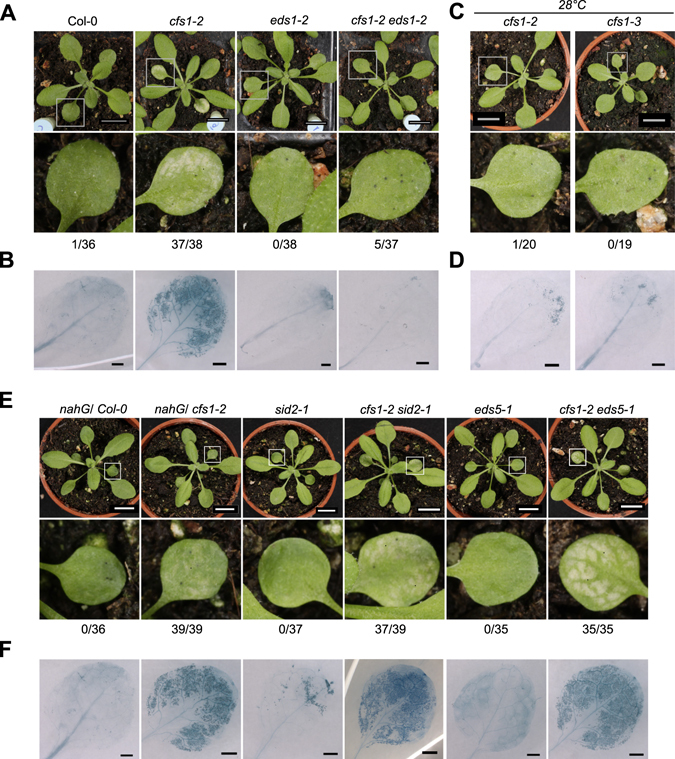
The HR-cell death phenotype in *cfs1* is dependent on EDS1-mediated ETI. (**A**) Three-week old Col-0, *cfs1-2*, *eds1-2*, *cfs1-2 eds1-2*. Square indicates leaf 1 or 2 that is digitally magnified below. Scale bars:1 cm. The values below each row represent the number of plants displaying lesions and total number of plants observed from one representative experiment, respectively. (**B**) Trypan blue staining of leaf 1 or 2 from a three-week old plant. The genotype for each image is indicated in the respective panel A. Scale bars: 1 mm. (**C**) *cfs1-2* and *cfs1-3* plants grown at 28 °C where ETI is rendered inactive. The values below each row represent the number of plants displaying lesions and total number of plants observed from one representative experiment, respectively. (**D**) Trypan blue staining of leaf 1 or 2 of *cfs1-2* and *cfs1-3* plants grown at 28 °C as in C. Scale bars: 1 mm. (**E**) Three-week old Col-0 plant expressing bacterial *nahG*, *cfs1-2* plant expressing bacterial *nahG*, *sid2-1*, *cfs1-2 sid2-1*, *eds5-1* and *cfs1-2 eds5-1*. Square indicates leaf 1 or 2 that is digitally magnified below. Scale bars: 1 cm. The values below each row represent the number of plants displaying lesions and total number of plants observed from one representative experiment, respectively. (**F**) Trypan blue staining of the first and second leaf from a three-week old plant. The genotype of each image is indicated in the respective panel C. Scale bars:1 mm.

In order to identify the underlying signal transduction pathway causing the *cfs1* cell death phenotype, we performed genetic analyses using mutants known to suppress cell death. We chose the upstream regulator *eds1* and genes that affect SA signaling. In *cfs1-2 eds1-2*, cell death was suppressed as judged by visual inspection and trypan blue staining (Fig. 3A,B). Because EDS1 mediates a branch of ETI that is dampened in ambient elevated temperature conditions^36^, we tested the connection between the *cfs1* phenotype and ETI by growing *cfs1* mutants at 28 °C. Under these conditions, *cfs1* mutants did not develop lesions indicating that cell death in *cfs1* requires ETI signal transduction (Fig. 3C,D). To test whether *cfs1* cell death requires SA signaling, we expressed the bacterial SA degrading enzyme *naphthalene hydroxylaseG* (*nahG*) in *cfs1-2* and generated double mutants of *cfs1-2* with the two SA deficient mutants, *sid2-1* and *eds5-1*. We found that the cell death phenotype was still present in *cfs1-2* expressing *nahG*, *cfs1-2 sid2-1* and *cfs1-2 eds5* (Fig. 3E,F).

Taken together, our results suggest that the *cfs1* lesion mimic phenotype depends on the EDS1-mediated signaling branch, but cell death initiation is independent of SA.

### Endosomal localization of CFS1 is mainly mediated by its SYLF domain

To understand the molecular function of CFS1, we performed cell biological and biochemical analyses. As FYVE domains are known to interact with the endosomally-enriched lipid PI3P^37^, we first analyzed the subcellular localization of CFS1. We co-expressed a 35S driven CFP-CFS1 fusion with markers for endosomes and Golgi complex in *Arabidopsis* protoplasts and observed co-localization only in the case of endosomes (Supplementary Figure S4). To reaffirm CFS1 localization on late endosomes, we generated transgenic lines expressing CFP-CFS1 under its native promoter and under double 35S promoter. Only the double 35S driven construct showed a fluorescence signal. The construct also rescued the cell-death phenotype of *cfs1* (Supplemental Figure S3B).

**Figure 4:**
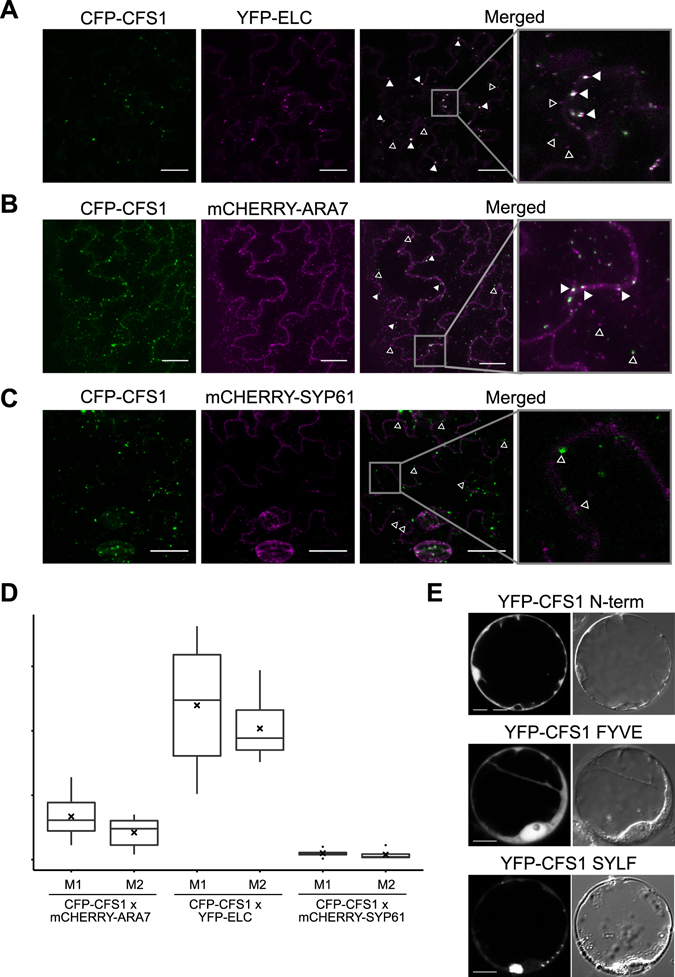
CFS1 SYLF domain mediates CFS1 localization on late endosome. (**A**) CFS1 co-localizes with the ESCRT-I component, ELC. Images are maximum Z-projection from representatives of two independent CFS1 transgenic lines. Arrowhead indicates co-localization. Scale bars: 25 µm. (**B**) CFS1 co-localizes with the late endosome marker, ARA7. Images are maximum Z-projection from representatives of two independent CFS1 transgenic lines. Arrowhead indicates co-localization. Scale bars: 25 µm. (**C**) CFS1 did not co-localize with the early endosome marker, SYP61. Images are maximum Z-projection from representatives of two independent CFS1 transgenic lines. Open arrowhead indicates non-colocalizing structure. Scale bars: 25 µm. (**D**) Manders’ overlap coefficient^67^ of transgenic lines co-expressing CFS1 and ARA7, ELC and SYP61 as shown in 4**A–C**. M1 indicates the fraction of CFS1 signal that overlapped with the marker of interest, and M2 indicates the fraction of the marker of interest that overlapped with CFS1 signal. Straight lines within boxes represent median, crosses represent mean value, and outliers are depicted as closed circles. Values were obtained from 5 cells originated from two independent transgenic lines. (**E**) CFS1 N-terminal fragments with and without FYVE domain show cytoplasmic localization (YFP-CFS1 N-term and YFP-CFS1 FYVE, respectively); whereas the SYLF domain localizes in punctuate structures. A bright field image is shown to the right of the corresponding images from fluorescent channel. Scale bars: 5 µm.

We crossed the CFP-CFS1 line to two transgenic lines expressing late endosomal markers, mCHERRY-ARA7 driven by a double 35S promoter and YFP-ELC driven by a 35S promoter. As early endosomal marker, we used mCHERRY-SYP61 driven by a double 35S promoter. We found partial co-localization in cases of ELC and ARA7 but no co-localization in the case of SYP61 (Fig. 4A–D). In the case of the ESCRT-I component ELC, a greater number of endosomes that were positive for CFS1 were also labeled by ELC while few of ELC-labeled endosomes that did not show CFS1 signal were also observed (Fig. 4A). By contrast, ARA7-labeled endosomes were found that showed co-labeling with CFS1 and were positive for CFS1 but not ARA7 and *vice versa*. (Fig. 4B). These experiments suggest that CFS1 is mainly localized at a subset of ESCRT-related MVBs.

Next, we addressed whether the FYVE domain of CFS1 is essential for the recruitment to MVBs. We analyzed the localization of individual CFS1 domains in Arabidopsis protoplasts (Figs 1 and 4E). Both, a YFP fusion with the CFS1 N-terminus and a YFP fusion with the FYVE domain displayed cytosolic localization (Fig. 4E). When analyzing the localization of YFP fused with SYLF, we observed fluorescence in punctate structures (Fig. 4E).

### Plant SYLF is a PI3P and actin binding domain

**Figure 5:**
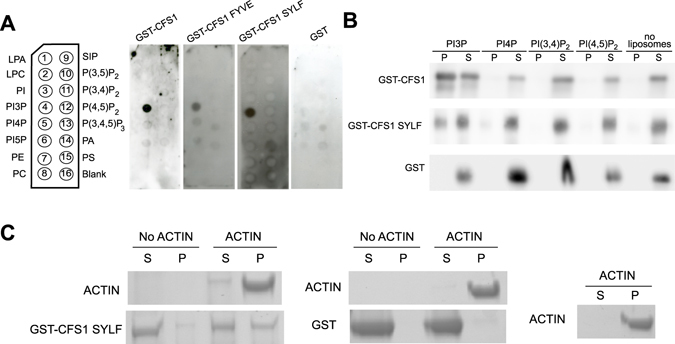
CFS1 is a PI3P- and actin-binding protein. (**A**) Lipid overlays dot blots showing that CFS1, its FYVE and SYLF domain specifically bind to PI3P. GST was used as a negative control. All stripes have the arrangement of lipid species shown on the left diagram. (**B**) Liposome sedimentation assays analyzed by SDS-PAGE and Coomassie staining show binding of CFS1 and its SYLF domain to PI3P. Liposomes contained PC/PE and 10% of the respective lipid species. GST and assays without liposomes were used as negative controls (P: pellet, S supernatant). Full-length blots are presented in Supplementary Figure S15. (**C**) Actin coesdimentation assays analyzed by SDS-PAGE and Coomassie staining show binding of GST-CFS1 SYLF domain to actin (P: pellet, S: Supernatant). Full-length gel is presented in Supplementary Figure S14.

To further examine the localization patterns of FYVE and SYLF, we studied the lipid-binding properties of both domains. We performed lipid overlay assays with bacterially expressed GST-CFS1 full-length, GST-CFS1 FYVE, and GST-CFS1 SYLF, using GST as negative control (Fig. 5A). All CFS1 fragments showed specific interaction with PI3P, whereas GST alone did not show any binding (Fig. 5A). To independently verify the PI3P binding of SYLF, we performed liposome cosedimentation assays with full-length GST-CFS1 and GST-CFS1 SYLF. Liposomes were prepared containing 50% PC, 40% PE and 10% of either PI3P, PI4P, PI3,4P_2_ or PI4,5P_2_. Controls included GST alone and assays without liposomes. Consistent with the lipid overlay assays, both the full-length protein and the SYLF domain specifically interacted with PI3P (Fig. 5B).

The analysis of the SYLF domain of yeast Ysc84p had revealed its interaction with actin^33^. Therefore, we performed an actin cosedimentation assay with GST-SYLF and GST-CFS1. GST-SYLF could be pelleted with pre-polymerized rabbit skeletal muscle actin (Fig. 5C) though the full-length protein did not display an interaction (Supplementary Figure S5). To test whether CFS1 is involved in the organization of the actin cytoskeleton, we inspected *cfs1* mutants for typical actin-related phenotypes. We analyzed trichome and pavement cell morphology and used Lifeact-eGFP to visualize possible defects of the actin cytoskeleton^38^. We found no differences between *cfs1* mutants and Col-0 in either of these experiments (Supplementary Figure S5B,C).

### *cfs1* mutants accumulate autophagosomes

To test whether CFS1 acts as VPS27 substitute in recruiting ESCRT-I to endosomes, we performed localization studies. As CFS1 specifically interacted with ELC, we tested the localization of YFP-ELC under the control of the 35S promoter in *cfs1* background. YFP-ELC was still localized in a punctate pattern as in wild type (Supplementary Figure S6), suggesting that CFS1 does not act as VPS27 and the CFS1/ELC interaction serves a different purpose. We further investigated whether CFS1, like the ESCRT-I components, is involved in PTI-mediated resistance against virulent *Pseudomonas syringae* pv. tomato (*Pto* DC3000)^39^. We therefore examined *cfs1* immunity to *Pto* DC3000 infection but found no differences compared to Col-0 (Supplementary Figure S7).

**Figure 6:**
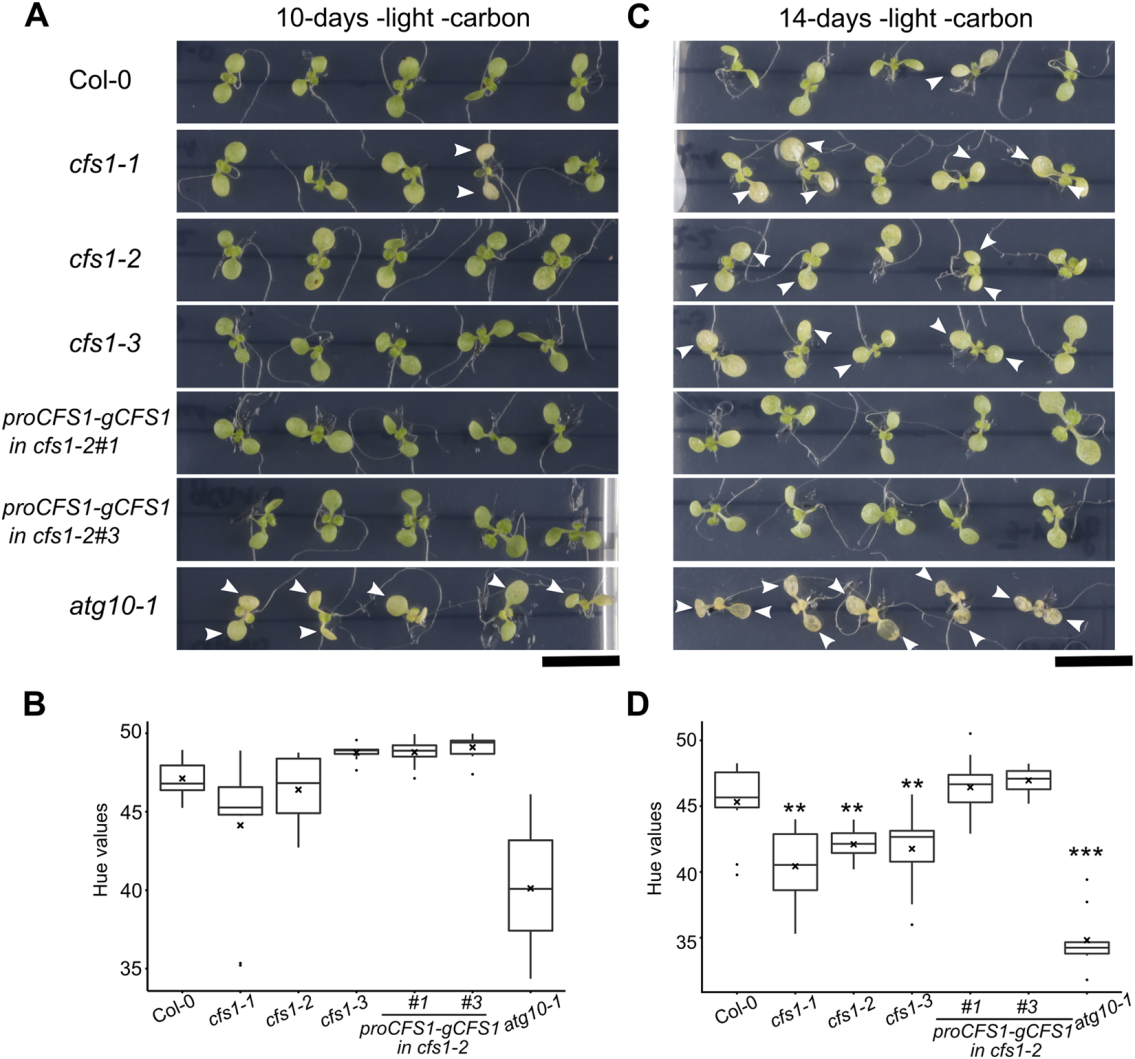
*cfs1* mutants are hypersensitive to carbon starvation. (**A**) 7-day old seedlings subjected to 10 days of carbon starvation show no difference in yellowing between the majority of *cfs1* mutants, Col-0 and the complemented lines (*proCFS1-gCFS1* in *cfs1-2*#1, #3). Arrowheads indicate yellowing in the cotyledons, and *atg10-1* plants were used as positive control. Scale bar: 1 cm. (**A**) Boxplots show the hue values of the cotyledons shown in (**A**). Straight line within the box represents median, mean value and outliers are depicted as cross and closed circles, respectively. (**C**) 7-day old seedlings subjected to 14 days of carbon starvation show cotyledon yellowing in the majority of *cfs1* mutants compared with Col-0 and the complemented lines. The genotypes of each row are arranged as in A. Arrowheads indicate yellowing in the cotyledons, and *atg10-1* plants were used as positive control. Scale bar: 1 cm. (**D**) Boxplots show the hue values of the cotyledons shown in (**C**). Straight line within the box represents median, mean value and outliers are depicted as cross and closed circles, respectively. Hue values of each genotype were compared to Col-0 using Wilcoxon rank sum test. Double asterisks indicated statistical significant difference at p ≤ 0.01 and triple asterisks at p ≤ 0.001.

One of the responses to signals generated from the EDS1 branch of ETI is autophagy induction and autophagosome formation^15^. Concomitantly, crosstalk between autophagosomes and ESCRT/MVBs has been described in plants^30–32^. To explore CFS1 connection to autophagy, we subjected *cfs1* mutants to carbon starvation using the autophagy mutant *atg10-1* as positive control^40^. After 10 days of carbon starvation, the majority of *cfs1* mutants, wild-type and the complemented lines did not display yellowing when it was readily observed in *atg10-1* (Fig. 6A,B). Yellowing of *cfs1* mutants appeared after 14 days of starvation whereas wild type and complemented lines were still green (Fig. 6C,D).

**Figure 7:**
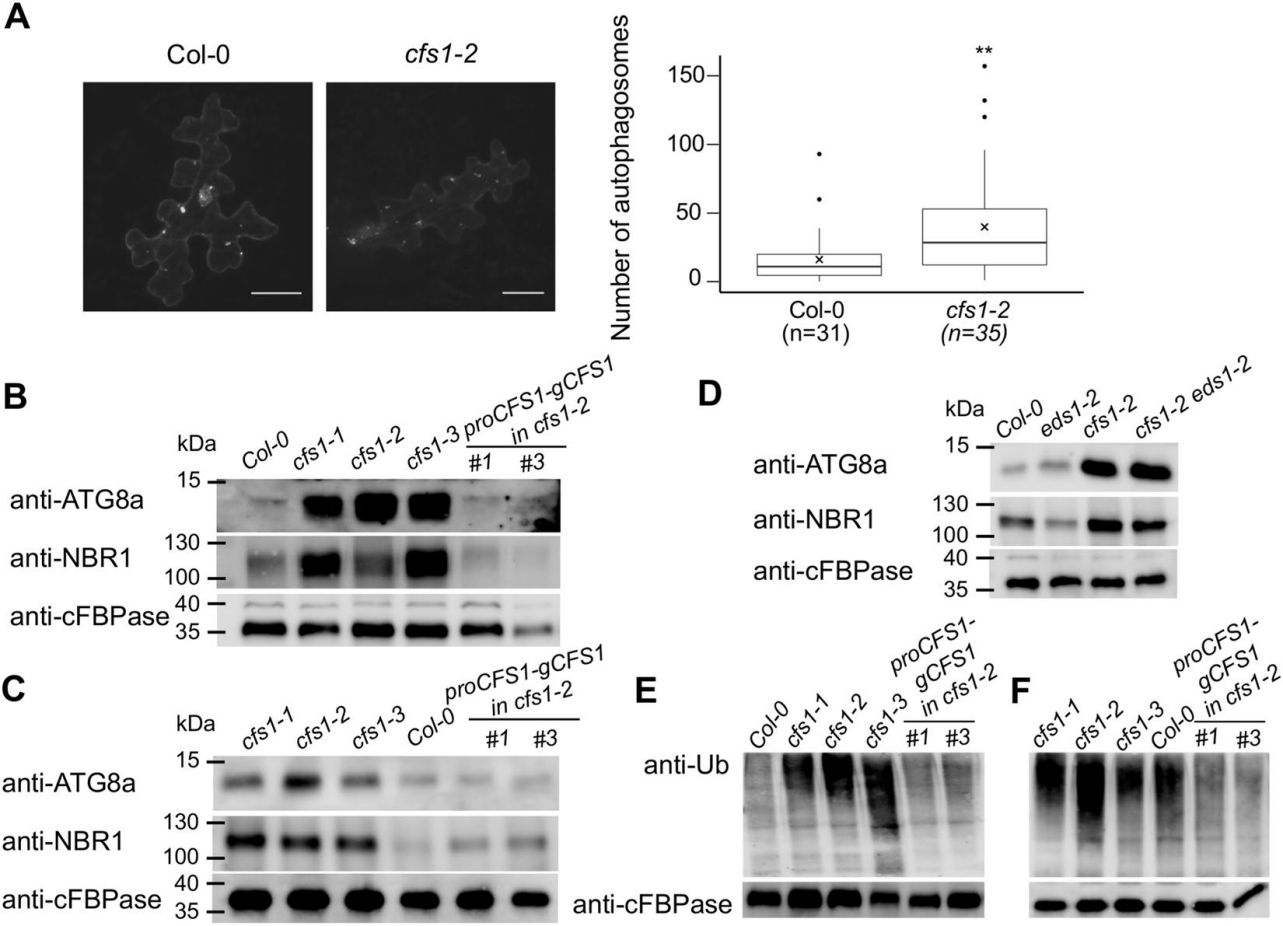
Autophagosomes are accumulated in *cfs1* mutant. (**A**) Transiently expressed YFP-ATG8a in *cfs1-2* epidermal leaf cells shows that YFP-ATG8a localized in punctate structures, indicating that autophagosomes can be formed. Scale bars are 25 µm. Boxplots show significantly higher number of autophagosomes in *cfs1-2* as compared to Col-0 (p ≤ 0.01 from Wilcoxon rank sum test). Straight lines within boxes represent median, crosses represent mean value, and outliers are depicted as closed circles. *n* indicates the number of cells used in the analysis. (**B**) Immunoblots of anti-ATG8a and anti-NBR1 reveal autophagosome accumulation in leaf 1 and 2 of three-week old *cfs1* mutants compared to Col-0 and the complemented lines of the same stage. Anti-cFBPase is used as loading control. Full-length blots are presented in Supplementary Figure S9. (**C**) Immunoblots of anti-ATG8a and anti-NBR1 reveal autophagosome accumulation in leaf 1 and 2 of two-week old *cfs1* mutants compared to Col-0 and the complemented lines of the same stage. Anti-cFBPase is used as loading control. Full-length blots are presented in Supplementary Figure S10. (**D**) Immunoblots of anti-ATG8a and anti-NBR1 reveal autophagosome accumulation in leaf 1 and 2 of two-week old *cfs1-2* and *cfs1-2 eds1-2* mutants compared to Col-0 and *eds1-2* of the same stage. Anti-cFBPase is used as loading control. Full-length blots are presented in Supplementary Figure S11. (**E**) Immunoblot of anti-Ub reveals substantial accumulation of ubiquitinated proteins in leaf 1 and 2 of three-week old *cfs1* mutants compared to Col-0 and the complemented lines of the same stage. Anti-cFBPase is used as loading control. Full-length blots are presented in Supplementary Figure S12. (**F**) Immunoblot of anti-Ub reveals accumulation of ubiquitinated proteins in leaf 1 and 2 of two-week old *cfs1* mutants compared to Col-0 and the complemented lines of the same stage. Anti-cFBPase is used as loading control. Full-length blots are presented in Supplementary Figure S13.

Deficiency in autophagy in *cfs1* could originate either from the failure to initiate or to degrade autophagosomes^41^. To address whether cfs1 mutants can still form autophagosomes, we transiently expressed YFP-ATG8a under control of the double 35S promoter. ATG8 localization changes from cytoplasmic to autophagosome localization upon autophagy initiation^41^ in *cfs1-2* as in *Col-0*, suggesting that autophagosomes can be initiated in *cfs1* mutants. To address whether autophagosome turnover might be impaired, we performed immunoblotting with antibodies against ATG8a and a plant autophagy cargo receptor, NEXT TO BRCA1 GENE 1 (NBR1) that are degraded in the vacuole in the course of autophagy^42^. We found that both ATG8a and NBR1 proteins accumulated in *cfs1* mutants showing lesions compared with Col-0 and the complemented lines of the same stage (Fig. 7B). Their transcript levels, by contrast, were comparable in all sample sets (Supplementary Figure S8) indicating that the disruption in autophagosome turnover in *cfs1* is not caused by enhanced expression. To investigate whether the disruption of autophagosome turnover in *cfs1* mutants is a consequence of ongoing cell death, we tested leaves from younger *cfs1* plants without lesions and from *cfs1-2 eds1-2* plants. Both accumulated ATG8a and NBR1 (Fig. 7C,D), demonstrating that CFS1 function in autophagosome turnover is independent of EDS1-mediated cell death. As autophagy has been proposed to contribute to HR through cellular homeostasis maintenance^43^, we assessed whether *cfs1* mutants display disturbed proteostasis. We found a substantial accumulation of ubiquitylated proteins in *cfs1* mutants showing lesions compared with Col-0 and the complemented lines of the same stage (Fig. 7E). The accumulation is less pronounced in younger stages (Fig. 7F). Overall, our results prompt us to hypothesize that CFS1 is a regulator of autophagic degradation thereby contributing to cellular proteostasis.

## Discussion

In this work, we have analyzed the function of CFS1, a previously uncharacterized Arabidopsis FYVE and a SYLF domain-containing protein. SYLF domains are conserved in fungi, vertebrates and plants. The best studied SYLF domain proteins are mammalian SH3YL1 and yeast Ysc84^33, 34^. In these proteins, the domain appears in combination with the protein-protein interaction domain SH3. Ysc84 and SH3YL1 interact with actin binding proteins and co-localize with cortical actin patches. Ysc84 has been shown to regulate early steps of endocytosis^33^, and SH3YL1 is localized at dorsal ruffles, actin-rich membrane regions with intense receptor endocytosis^34^. Both proteins appear to contribute to the integration of F-actin and membrane morphology^44^. Accordingly, their SYLF domains have been shown to bind to phospholipids^34, 44^, and in case of Ysc84 to actin^33^. Biochemically, CFS1 shows similarities to the behavior of Ysc84. It can bind to phospholipids, and only the isolated domain can bind to actin (Fig. 5A–C). However, the phospholipid binding specificity differs, reflecting the different localizations in the endomembrane system. Whereas Ysc84 and SH3YL1 bind to plasma membrane enriched PIs^34, 44^, CFS1 specifically interacts with PI3P (Fig. 5A,B) that is enriched in the endosomal and autophagosomal membrane. Interestingly, the localization of Ysc84 is not dependent on this interaction, but instead on protein interactions of the SH3 domain^33^. By contrast, the CFS1 SYLF domain appears to directly determine CFS1 localization (Fig. 4C).

The SYLF domain has also been termed YAB (Ysc84 ACTIN BINDING) domain because it confers the yeast protein Ysc84 binding to actin. YAB actin binding can only be observed with the isolated domain, but not the full-length protein. In full length Ysc84, actin can only bind when the interaction partner Las17p is associated with the adjacent SH3 domain^33^. Phospholipid binding inhibits the actin interaction^44^. CFS1 shows a similar actin binding activity with the isolated SYLF domain, but not with the complete protein (Figs 4F and S5A). As CFS1 contains an adjacent FYVE domain that also binds to PI3P, it is possible that actin binding of its SYLF domain is regulated by membrane association and lipid interaction of the FYVE domain. Alternatively, the FYVE domain could contribute to the membrane association of CFS1 in a cooperative manner as observed for other proteins containing multiple lipid binding domains^45^.

Genetically, CFS1 acts as an autophagy regulator and repressor of cell death (Figs 2, 6 and 7). Both processes are tightly connected. Autophagy has been reported to participate in both pro-death and pro-survival events in *Arabidopsis*, depending on the plant stage and circumstances^46^. In the event of pathogen infection, HR can be suppressed in young *atg* mutants^15, 47^ but enhanced in older *atg* mutants^48^. In this respect, two scenarios of how autophagy contributes to cell death have been proposed. First, autophagy regulates HR-cell death directly through a branch of the SA signaling cascade^48^ in an age- or tissue-dependent manner. Second, autophagy contributes to HR by maintaining cellular homeostasis. In this scenario, the inconsistent HR-cell death manifestation in older and younger *atg* mutants can be explained by longer SA accumulation and endoplasmic reticulum (ER) stress that build up from cellular homeostasis imbalance in older *atg* plants^43^. Alternatively, a combination of these scenarios has been proposed. In young plants, autophagy mediates vacuolar loading resulting in HR-cell death, thereby acting as cell death executioner. In aging plants, the autophagy pro-death role is masked by an increasing amount of damaged proteins or organelles^47^. In the *cfs1* mutants, misregulation of autophagy seems to be the primary defect as the accumulation of the autophagy markers ATG8 and NBR1 can already be observed in young *cfs1* plants not yet exhibiting cell death and in the *cfs1 eds1* double mutant (Fig. 7C,D). We also found that the level of ER-stress marker genes was comparable to wild type plants (data not shown), and that the cell death initiation in *cfs1* is SA-independent (Fig. 3C,D). The enhanced protein levels of ATG8a and NBR1 in *cfs1* (Fig. 7C,D) and their unchanged transcript levels (Supplementary Figure S7) suggest that the disruption of autophagosome turnover results from reduced autophagosome degradation rather than enhanced initiation. Consistent with that, a significantly higher although highly variable number of punctuate structures representing autophagosome membrane protein were found in the mutant (median = 11, $\bar{x}=16.2$ in Col-0 and median = 28.5, $\bar{x}=39.9$ in *cfs1-2*; Fig. 7A). As it has been recently shown that autoimmunity can result from the detection of modified NB-LRR proteins^49^, it is tempting to speculate that CFS1 might be guarded by an EDS1-dependent TIR-NB-LRR, and the lack of CFS1 results in false initiation of EDS1-mediated signaling. Alternatively, components of the autophagy machinery can be targeted by pathogen effectors in tobacco^50^, autoimmunity in *cfs1* could also result from an imbalance in cellular homeostasis. This scenario is supported by the accumulation of ubiquitylated proteins in *cfs1* (Fig. 7E,F) that possibly reflects the disturbed autophagosome turnover.

At first glance, actin binding of CFS1 seems to support an early function of CFS1 in autophagosome formation. In mammals, actin is involved in early events of phagophore formation in starvation-induced condition and depolymerization of actin leads to inhibition of autophagosome formation^51^. Furthermore, Arabidopsis NAP, a component of the SCAR/WAVE complex acts in an early stage of autophagosome formation, and autophagosome initiation is reduced in the mutant^52^. However, a recent report in mammals also places actin at a later stage in autophagosomal fusions with lysosomes during selective autophagy of protein aggregates. Loss of cortactin or inhibition of actin polymerization blocks the fusion of autophagosomes with lysosomes^53^. In mammals, it has also been shown that loss of the actin-dependent motor molecule MYOSIN VI not only leads to autophagosome accumulation, but MYOSIN VI also interacts with an ESCRT-0 related component of the ESCRT machinery, TOM1, which suggests a role of the actin cytoskeleton for the fusion of autophagosomes with ESCRT containing endosomes^54^. We have found binding of CFS1 to the ESCRT-I component ELC (Supplementary Figure S2B,C), but CFS1 was not necessary for the localization of ELC to endosomes (Supplementary Figure S6) unlike the yeast or mammal ESCRT-0 component^24^. Together with the finding that CFS1 co-localized with a subset of ELC-positive endosomes (Fig. 4A), we hypothesize that CFS1 does not act as classical ESCRT-0 component but might be involved in fusion events between autophagosomes and ESCRT-positive late endosomes, thereby affecting autophagosome degradation and protein homeostasis. In the mutant, the disturbed protein homeostasis could in turn triggers EDS1-mediated cell death.

## Methods

### Plant material, growth conditions, transfection procedures

T-DNA alleles SALK_018265 (*cfs1-1*), SALK_024058 (*cfs1-2*) and SALK_068647 (*cfs1-3*) were obtained from the Nottingham *Arabidopsis* Stock Centre, Nottingham, UK^55^. The transgenic line *nahG*, *eds1-2*, *eds1-5* and *sid2-1* have been previously described^7, 10, 56, 57^.

Plants were grown under long day non-sterile greenhouse conditions at 22 °C unless otherwise stated.

For carbon starvation, seedlings were germinated and grown on solid MS medium supplemented with 1% sucrose for 7 days, then transferred to solid MS medium without sucrose and grown in darkness for the indicated period of time. Chlorophyll loss was quantified by converting high resolution photographs of the seedlings to the HSB color mode and determining the hue values of the cotelydons with the imageJ measuring function^58^.

Transgenic plants were created by the floral dip method using the *Agrobacterium* strain GV3101 pMPRK90^59^. Transient expression of *Arabidopsis* leaves were achieved by biolistic transfection using a PDS-1000 He System (BioRad) as described earlier^60^ with following modifications: 0.15–0.3 mg of gold particles were coated with 400–600 ng of DNA, 900 psi rupture discs were used for bombardment. Samples were incubated overnight in the dark before microscopic observation.

Protoplasts were isolated and transformed as previously described^61^.

### Constructs and molecular biology

All constructs used for Y2H, fluorescent microscopy and protein expression were PCR-amplified and introduced to donor and destination vectors using Gateway BP and LR clonase enzyme mix according to the manufacturer’s instructions (Invitrogen). After BP reaction, PCR fragments in pDONR201 or pDONR207 were sequenced.

The CFS1 genomic construct was amplified from Col-0 genomic DNA in three amplicons that were separately subcloned into pJET1.2 via the EcoRV site using CloneJet PCR cloning kit, Fermentas), sequenced then ligated to the pAMPAT vector via AscI and ApaI sites.

All primers, vectors and AGI codes of genes used in this study are listed in Supplementary Tables 1 and 2.

### Light microscopy, TEM and Confocal Laser Scanning Microscope

Leaf 1 or 2 of plants at 8-leaves stage were stained with trypan blue as previously described^62^ and imaged with a Leica MZ16F stereo microscope.

For TEM and semi-sections, 6–10 leaves of Col-0 and the three *cfs1* alleles were fixed for 4 hours at room temperature (2.5% Glutaraldehyde, 2% paraformaldehyde, 0.08 M HEPES-NaOH, pH 7.0). After postfixation (1% OsO_4_ in 80 mM HEPES-NaOH pH 7.0, 2 h) at room temperature, samples were treated with 1% uranylacetate overnight, in darkness at 4 °C, dehydrated by aceton washes on ice, infiltrated in SPURR resin and polymerized at 70 °C overnight^63^. 1 µM semi-thin sections and 60–80 nm ultra-thin sections were prepared with a Leica EM UC7 microtome equipped with a DiATOME diamond knife at 45° angle. Semi-thin sections were stained with a 1% toluidine blue in 1% Borax 1% Pyronin G solution (5:1). Ultrathin sections were mounted on pioloform covered 1 mm copper grids, contrasted with Reynold’s lead citrate for 1 min^64^ and observed with a Philips CM10 TEM. Micrographs were taken with Orius SC200W CCD camera equipped with the DigitalMicrograph software (Gatan Inc).

Confocal images were acquired using a Leica TSC SPE or SP8. ImageJ 2.0^65^ was used to obtain maximum projection images, assign color lookup tables and analyze the number of punctuate structures. Colocalization analysis was performed using JACoP plug-in available in ImageJ 2.0 with maximum Z-projection images and threshold manually adjusted.

### RNA preparation and quantitative RT-PCR

Leaves 1 and 2 were collected from four-week old plants and frozen in liquid nitrogen. Total RNA was extracted using RNeasy Plant Mini kit (QIAGEN). 0.7 µg of total RNA were reverse transcribed with an oligo(dT) primer and SuperScript III reverse transcriptase (Invitrogen) following the manufacturer’s instructions. Subsequent quantitative real-time PCR was performed with Power SYBR Green PCR Master Mix (Applied Biosystems) in an Applied Biosystems 7300 Real-Time PCR System. All reactions were amplified for 40 cycles using a two-step protocol at 95 °C for 15 s and 60 °C for 1 min followed by a dissociation step at 95 °C for 15 s, 60 °C for 20 s then 95 °C for 15 s, 60 °C for 15 s. Values obtained were analyzed using R statistical programming language 3.2.1.

### Protein expression, lipid overlay, liposome and actin cosedimentation

CFS1 protein and its fragments were expressed as N-terminal GST-fusions (pGEX2T-MGW) in ArcticExpress cells (Agilent Technologies). An overnight culture derived from a single colony was used in a 1:100 dilution as a starter. The main culture was grown for 3 h at 30 °C and shaking at 220 rpm. After induction with 1 mM IPTG temperature was set to 12 °C. Cells were harvested after 24 h, incubated with 100 µg/ml lysozyme for 20 min and lysed 2 times by ultrasound treatment (1 min, continuous pulse, 60% output, Sonoplus, Bandelin) in lysis buffer (50 mM HEPES-NaOH pH 8.0, 500 mM NaCl, 5 mM DTT, 1× Complete EDTA-free tablet (Roche)). The raw extract was centrifuged at 3200 g, 4 °C for 20 min. The supernatant was supplemented with 4% Triton X-100 and incubated with Glutathione Sepharose 4 Fast Flow (GE Healthcare; 0.5 ml per 1 l culture) for two hours at 4 °C. After washing with 10 ml washing buffer (50 mM HEPES-NaOH, pH 8.0, 500 mM NaCl) the protein was eluted with elution buffer (50 mM HEPES-NaOH pH 8.0, 500 mM NaCl, 40 mM reduced glutathione). Elution fractions were pooled, concentrated in amicons (Millipore) and transferred to 10 mM TRIS-HCl pH 7.5, 100 mM NaCl buffer with a Microdialyser (Scienova) following the manufacturer’s instructions.

Lipid overlay assays were performed with GST-fusions of the respective protein fragments using Echelon strips according to the manufacturer’s instructions.

Phosphoinositide containing liposomes (Avanti Polar Lipids) were prepared as previously described^66^ and passed 17 times through a 100 nm polycarbonate filter (Avanti Polar Lipids). After centrifugation of the protein at 45,000 g, for 15 min, at 4 °C, 18 µl of liposomes were incubated with 2 µM protein in liposome buffer (10 mM TRIS-HCl pH 7.5, 100 mM NaCl, 0.5 mM DTT) for 15 min followed by centrifugation as above.

Western blots were performed in semi-dry condition (BioRad). The presence of protein was detected using the following primary and secondary antibodies: anti-GST goat polyclonal (GE Healthcare), anti-ATG8a rabbit polyclonal (AB77003, ABCAM), anti-NBR1 rabbit polyclonal (AS14 2805, Agrisera), anti-cFBPase rabbit polyclonal (AS04 043, Agrisera), donkey anti-goat IgG-HRP (Santa Cruz Biotech), goat anti-rabbit HRP (Sigma-Aldrich), anti-Ub(P4D1) (Santa-Cruz).

Actin cosedimentation assays have been performed as previously described^33^.

### Data availability

The datasets generated during and/or analysed during the current study are available from the corresponding author on reasonable request.

## Electronic supplementary material

Supplemental figures
