# Supplementary material for: Disruption of the plant-specific CFS1 gene impairs autophagosome turnover and triggers EDS1-dependent cell death
Source: Sci Rep. 2017 Aug 17;7:8677. doi: 10.1038/s41598-017-08577-8 (PMC5561093; doi:10.1038/s41598-017-08577-8)
Supplement: Supplementary file 1 — Supplemental figures [file 41598_2017_8577_MOESM1_ESM.pdf]

- Supplemental Material -

Disruption of the plant-specific *CFS1* gene impairs autophagosome turnover and triggers EDS1-dependent cell death

Arpaporn Sutipatanasomboon[1], Stefanie Herberth[1,], Ellen G. Alwood[2], Heidrun Häweker[3], Britta Müller[1], Mojgan Shahriari[1,4], Anke Y. Zienert[5], Birger Marin[6], Silke Robatzek[3], Gerrit J. K. Praefcke[5,6], Kathryn R. Ayscough[2], Martin Hülskamp[1,#] and Swen Schellmann[1,#]

[1] Botanik III, Biocenter, Universtiy of Cologne, Zülpicher Str. 47B, 50674 Cologne, Germany,

[2] Department of Biomedical Science, The University of Sheffield, Western Bank Sheffield S10 2TN, United Kingdom

[3] The Sainsbury Laboratory, Norwich Research Park, Norwich NR4 7UH, United Kingdom

[4] Institut für Biologie II, University of Freiburg, Schänzlestrasse 1, 79104 Freiburg i. Br., Germany

[5]Institut für Genetik, Universtiy of Cologne, Zülpicher Str. 47A, 50674 Cologne, Germany

[6] Botanik I, Biocenter, Universtiy of Cologne, Zülpicher Str. 47B, 50674 Cologne, Germany

[7] Division of Haematology / Transfusion Medicine, Paul-Ehrlich-Institut, Federal Institute for Vaccines and Biomedicines, Paul-Ehrlich-Str. 51-59, 63225 Langen, Germany

Corresponding authors[#]: Swen Schellmann (swen.schellmann@uni-koeln.de) and Martin Hülskamp (martin.huelskamp@uni-koeln.de)

Supplementary Figures

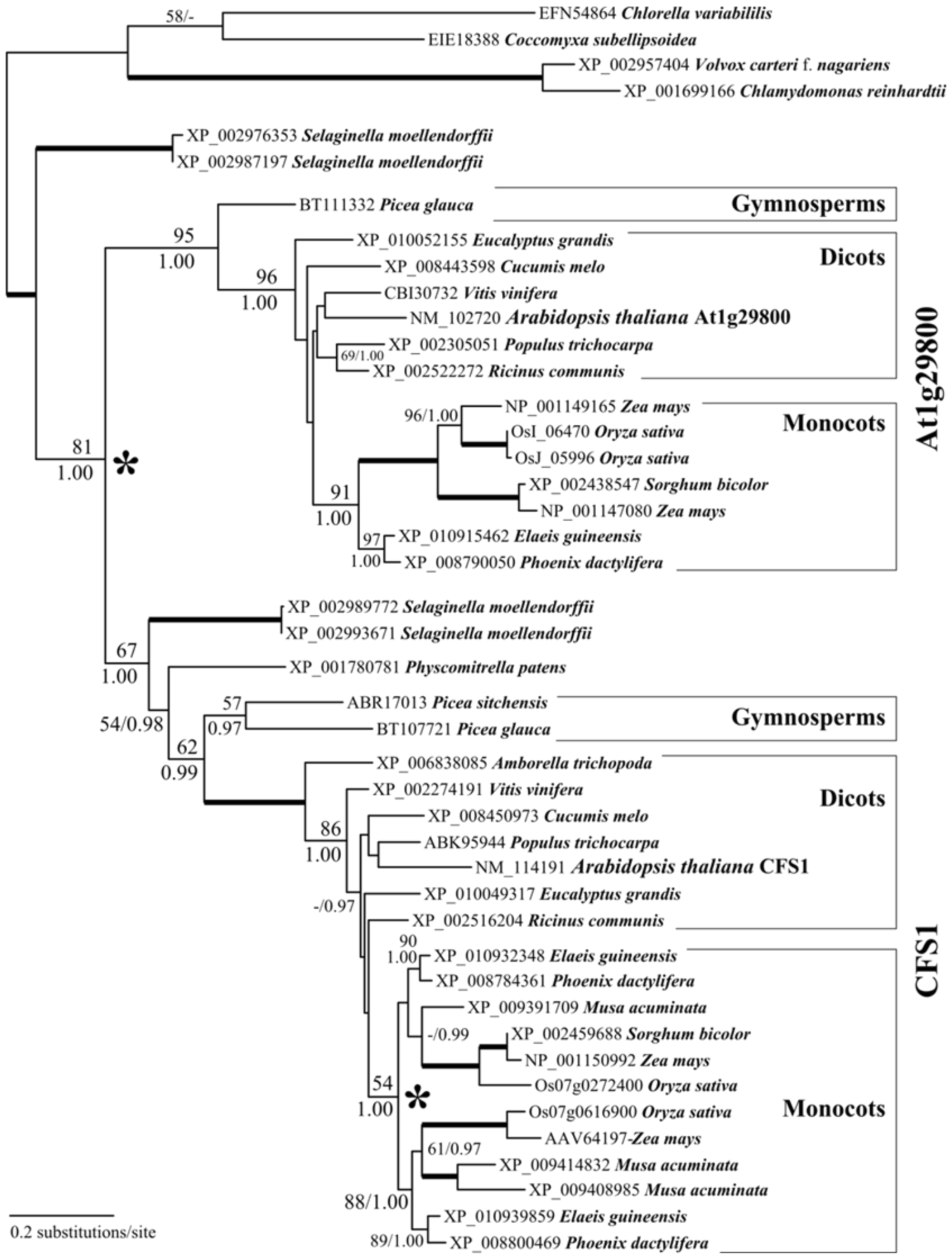

Supplementary Figure S1: CFS1 and At1g29800 are evolutionary conserved in the plant kingdom. Phylogenetic tree of CFS1 and At1g29800 homologs from 44 taxa. Bootstrap values are shown above, Bayesian posterior probabilities below the branches. Bold branches indicate maximal values of both support values. Green algae are shown as outgroup. Asterisks mark the most likely gene duplication events. One diversification has occurred before the origin of seed plants, a second among monocots within the CFS1 subtree.

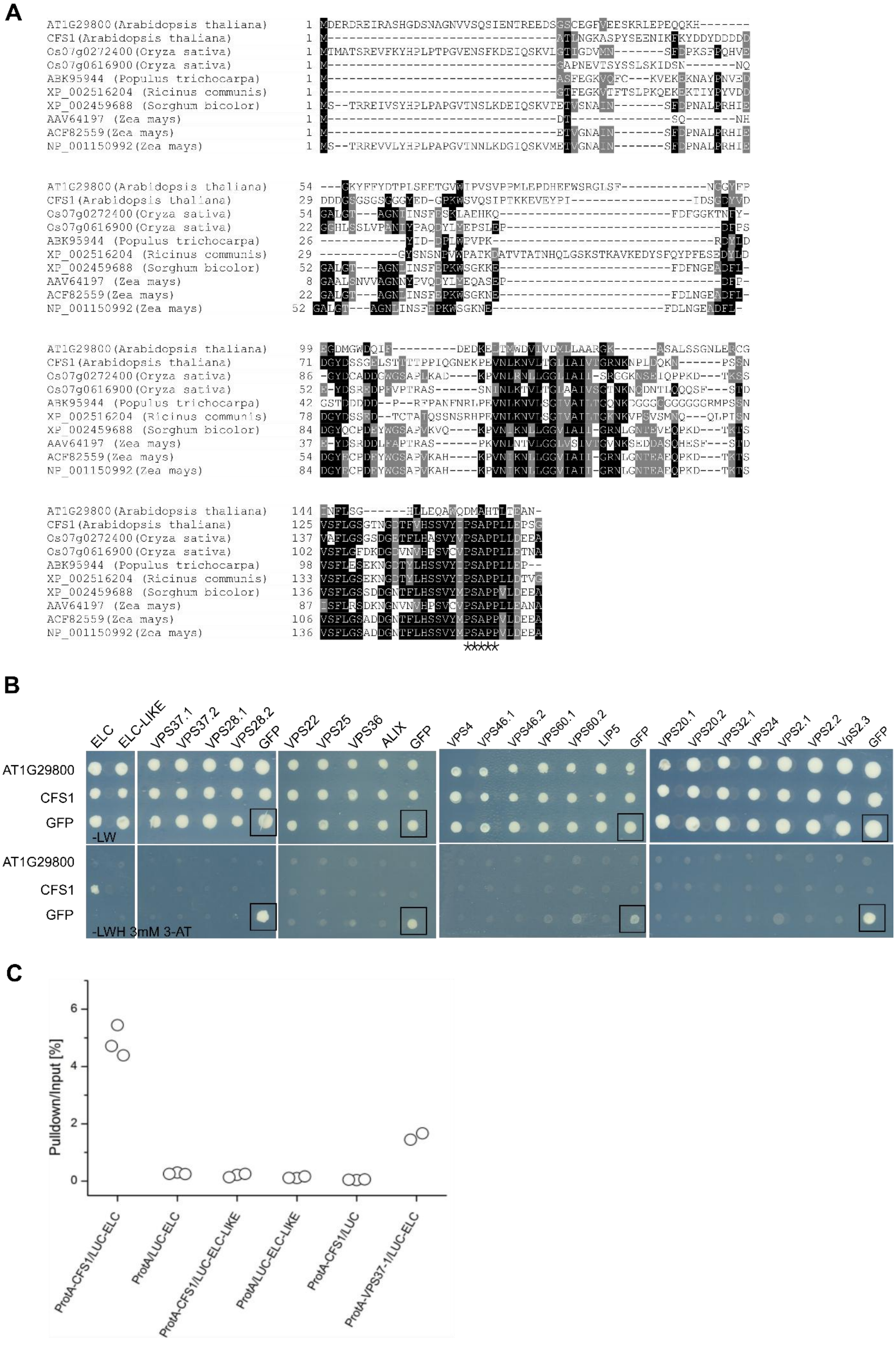

A
AT1G29800(Arabidopsis thaliana)
CFS1(Arabidopsis thaliana)
Os07g0272400(Oryza sativa)
Os07g0616900(Oryza sativa)
ABK95944 (Populus trichocarpa)
XP_002516204 (Ricinus communis)
XP_002459688 (Sorghum bicolor)
AAV64197 (Zea mays)
ACF82559(Zea mays)
NP_001150992(Zea mays)
B
ELC
ELC-LIKE
VPS37.1
VPS37.2
VPS28.1
VPS28.2
GFP
VPS22
VPS25
VPS36
ALIX
VPS4
VPS46.1
VPS46.2
VPS60.1
VPS60.2
LIP5
VPS20.1
VPS20.2
VPS32.1
VPS24
VPS2.1
VPS2.2
VpS2.3
AT1G29800
CFS1
-LW
-LWH 3mM 3-AT
C
Pulldown/Input [%]
ProtA-CFS1/LUC-ELC
ProtA/LUC-ELC
ProtA-CFS1/LUC-ELC-LIKE
ProtA/LUC-ELC-LIKE
ProtA-CFS1/LUC
ProtA-VPS37-1/LUC-ELC

Supplementary Figure S2: Only CFS1 contains a conserved PSAPP motif in the N-terminus and interacts with ELC.

A Alignment of amino acid sequences of At1g29000 N-terminus with N-termini of CFS1 and CFS1-like proteins. Conserved amino acids are shown in black, amino acids with similar chemical properties are shown in grey. Asterisks mark the conserved PSAPP motif**.**

B Pairwise Y2H with At1g29800, CFS1 and Arabidopsis ESCRT components. CFS1, At1g29800 and GFP were used as baits, and ESCRT components were used as preys. For each experiment the transformation controls are shown in the top panel, the interaction plates are shown in the bottom panel. Snf1 and Snf4 were used as positive controls (box; Fields and Song, 1989). This experiment was performed as previously described (Shahriari et al., 2011).

C CFS1 interaction with ELC was confirmed in a LUMIER assay as previously described (Blasche and Koegl, 2013) with modifications (Pesch et al., 2013). CFS1 was fused to protein A, ELC and ELC-LIKE were used as luciferase fusions. One data point represents percentage of luciferase activity in the pulldown fraction compared to the input from one independent assay. The previously identified VPS37 and ELC interaction (Spitzer et al., 2006) was used as a positive control.

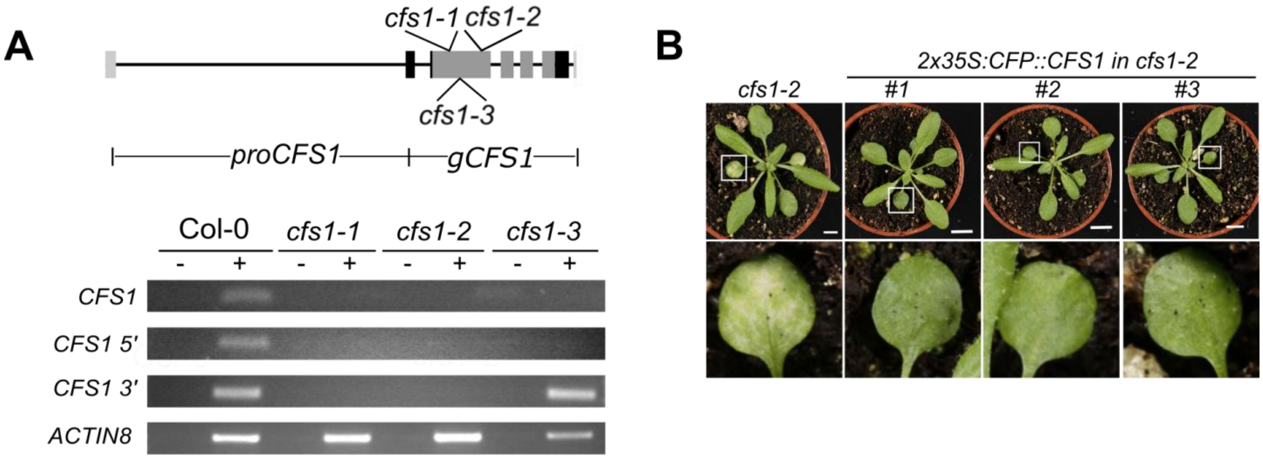

Supplementary Figure S3:

A Diagram depicting the *CFS1* locus, including the 3'UTR of the preceding gene and the 5'UTR of the following. Exons are shown as boxes. The CDS region is shown in grey, non-coding regions in black. Insertion locations of the three T-DNA alleles are indicated as open triangles. *CFS1* expression in *cfs1* mutants was quantified using semi-quantitative RT-PCR (30 amplification cycles) with three primer pairs: flanking the inserted region (CFS1), 5‘ of the insertion site (CFS1 5') and3' (CFS1 3') of the insertion site. *ACTIN8* primers were used as control. (+) and (-) indicate the presence and absence of reverse transcriptase during cDNA synthesis.

B CFP-CFS1 driven by a double 35S promoter in *cfs1-2* can complement the cell death phenotype when compared to *cfs1* mutant of the same stage, demonstrating that the overexpression CFP-CFS is function. Each picture represents a T3 individual from three independent transgenic lines. Boxed leaves are shown as magnifications in the lower panel. Scale bars: 1 cm.

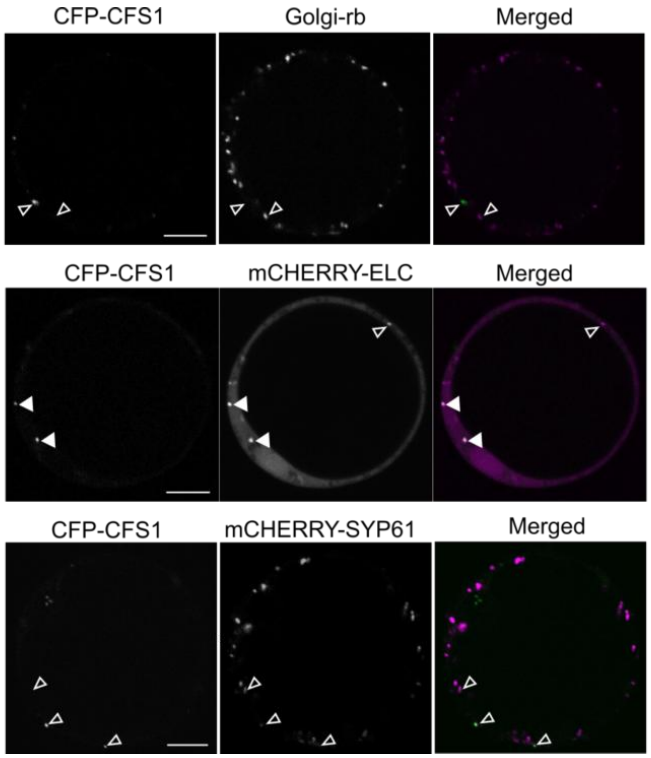

Supplementary Figure S4: CFS1 co-localizes with ELC but not with Golgi or trans-Golgi network markers. Transient co-expression of CFS1 and Golgi marker, Golgi-rb (Nelson et al., 2007), CFS1 and ELC (Spitzer et al., 2006) in protoplasts, and CFS1 and SYP61 (Herberth et al., 2012) in protoplasts. Closed arrowheads show co-localization; open arrowheads show non-localization. In the overlay pictures of the protoplast assays, CFP fluorescence appears in green, mCherry fluorescence in magenta. Scale bars: 7.5 µm

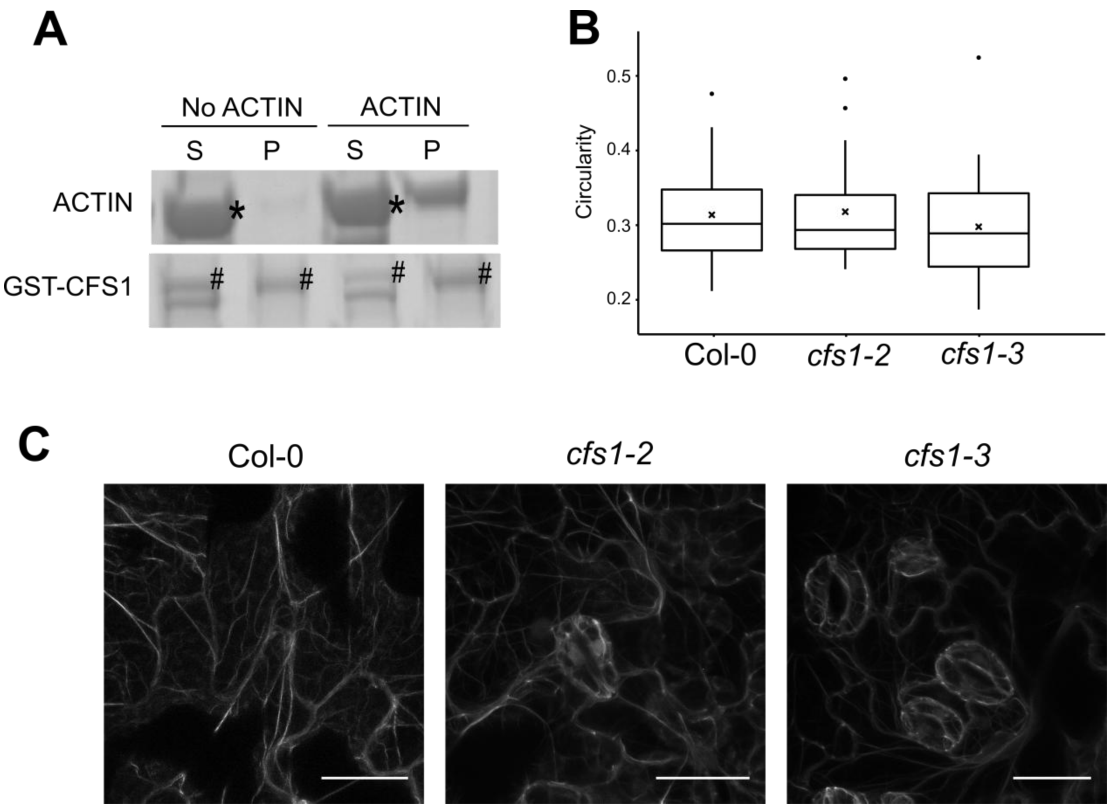

Supplementary Figure S5: *cfs1* mutants show no an apparent actin-deficiency phenotype.

A Actin cosedimentation assays of GST-fusions of full-length CFS1 revealed that full-length CFS1 cannot bind to actin. All images show Coomassie staining. (S: Supernatant, P: pellet. # contamination band.)

B No marked difference in the circularity of pavement cells in Col-0, *cfs1-2* and *cfs1-3* plants as revealed by ANOVA test ($p \geq 0.05$). Straight lines within boxes represent median, crosses represent mean value, and outliers are depicted as closed circles. Data were from 20 cells obtained from five plants.

C Epidermal leaf cells from transgenic plants expressing Lifeact-eGFP in Col-0, *cfs-2* and *cfs1-3* plants showed no apparent deviation of the actin cytoskeleton in *cfs1*. Scale bars: 25 µm.

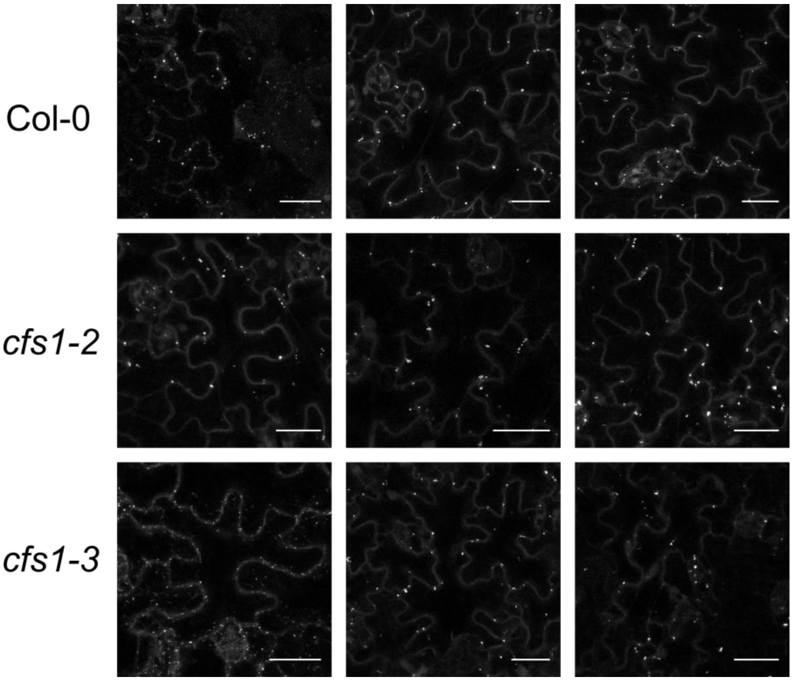

Supplementary figure S6: Lack of CFS1 does not delocalize ELC. Epidermal leaf cells of transgenic plants expressing YFP-ELC under the control of a 35S promoter show no delocalization of ELC in *cfs1-2* nor *cfs1-3*. Images are maximum Z-projection from representatives of three independent transgenic lines in each genotype background. Scale bars: 25 µm.

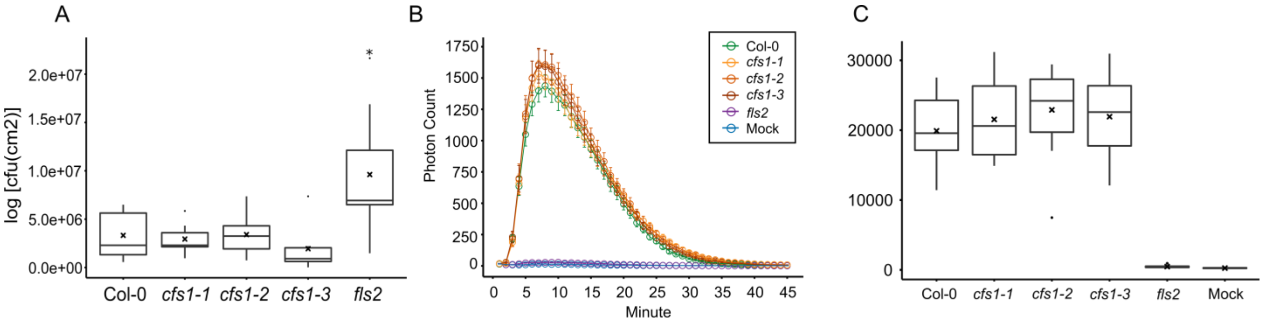

Supplementary figure S7: *cfs1* has no altered sensitivity to *Pto* DC3000 infection

A Bacterial counts following *Pto* DC3000 bacteria infection in Col-0 and *cfs1* mutants. Values are were obtained from 6-8 replicates and plotted in logarithmic scale. Straight lines within boxes represent median, crosses represent mean value, and outliers are depicted as closed circles. Only bacterial counts in *fls2* were significantly different from Col-0 (Wilcoxon rank sum test at $p \leq 0.05$).

B ROS development over 45 minutes treatment with 100 nM flg22 peptide. Error bars represent standard deviations of six replicates. Mock test and *flagellin sensing 2* (*fls2*) mutant were used as negative control in (Zipfel et al. 2004).

C Total photo count from ROS development depicted in B. Straight lines within boxes represent median, crosses represent mean value, and outliers are depicted as closed circles.

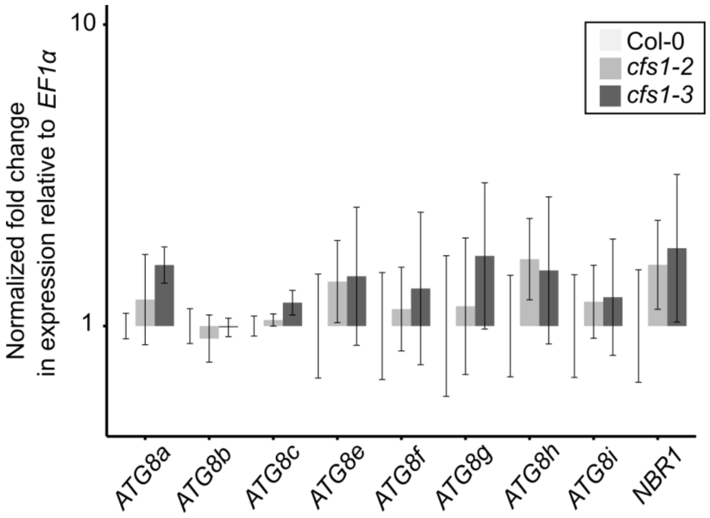

Supplementary figure S8: ATG8 genes are not upregulated in *cfs1* mutants. ATG8 genes and NBR1 transcript expression in *cfs1-2* and *cfs1-3* compared with Col-0. Values are plotted in log10 exponential scale. Error bars represent the standard error of three biological replicates with two technical replicates. No significant difference was detected when analyzed with ANOVA ($p \geq 0.05$).

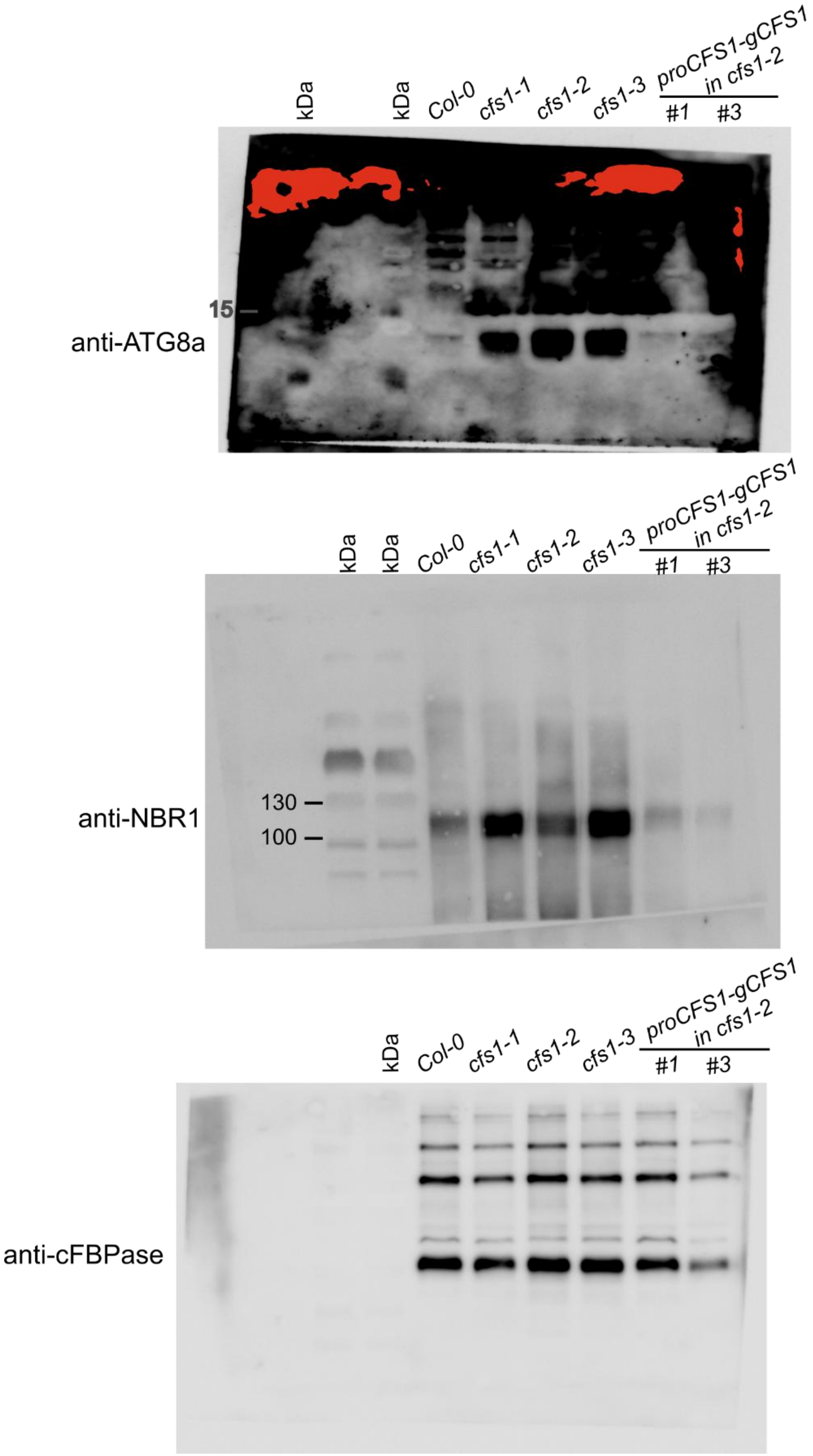

Supplementary figure S9: Full-length Immunoblots of anti-ATG8a and anti-NBR1 reveal autophagosome accumulation in leaf 1 and 2 of three-week old cfs1 mutants compared to Col-0 and the complemented lines of the same stage. Anti-cFBPase is used as loading control.

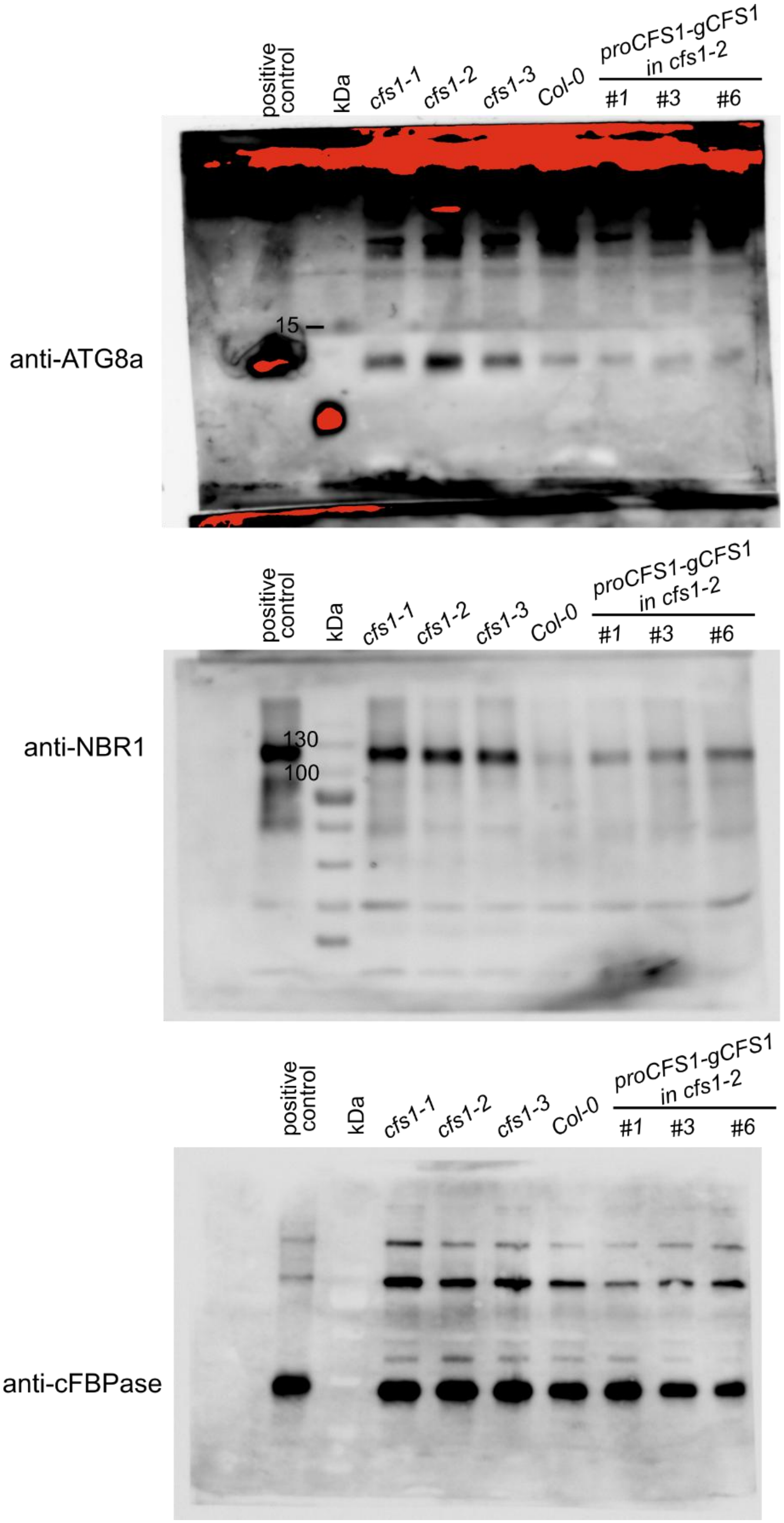

Supplementary figure S10: Immunoblots of anti-ATG8a and anti-NBR1 reveal autophagosome accumulation in leaf 1 and 2 of two-week old *cfs1* mutants compared to Col-0 and the complemented lines of the same stage. Anti-cFBPase is used as loading control.

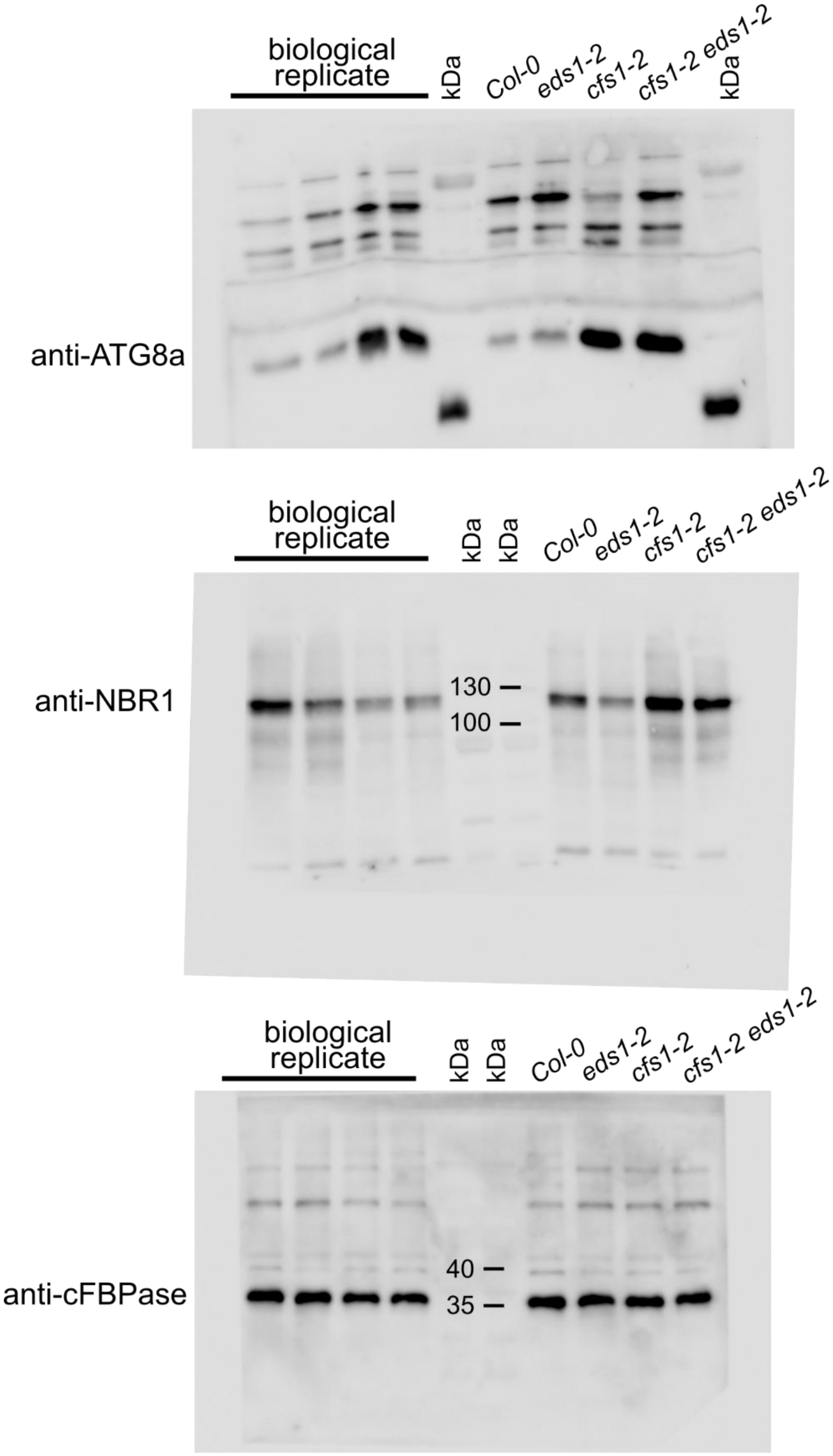

Supplementary figure S11: Immunoblots of anti-ATG8a and anti-NBR1 reveal autophagosome accumulation in leaf 1 and 2 of two-week old *cfs1-2* and *cfs1-2 eds1-2* mutants compared to Col-0 and *eds1-2* of the same stage. Anti-cFBPase is used as loading control.

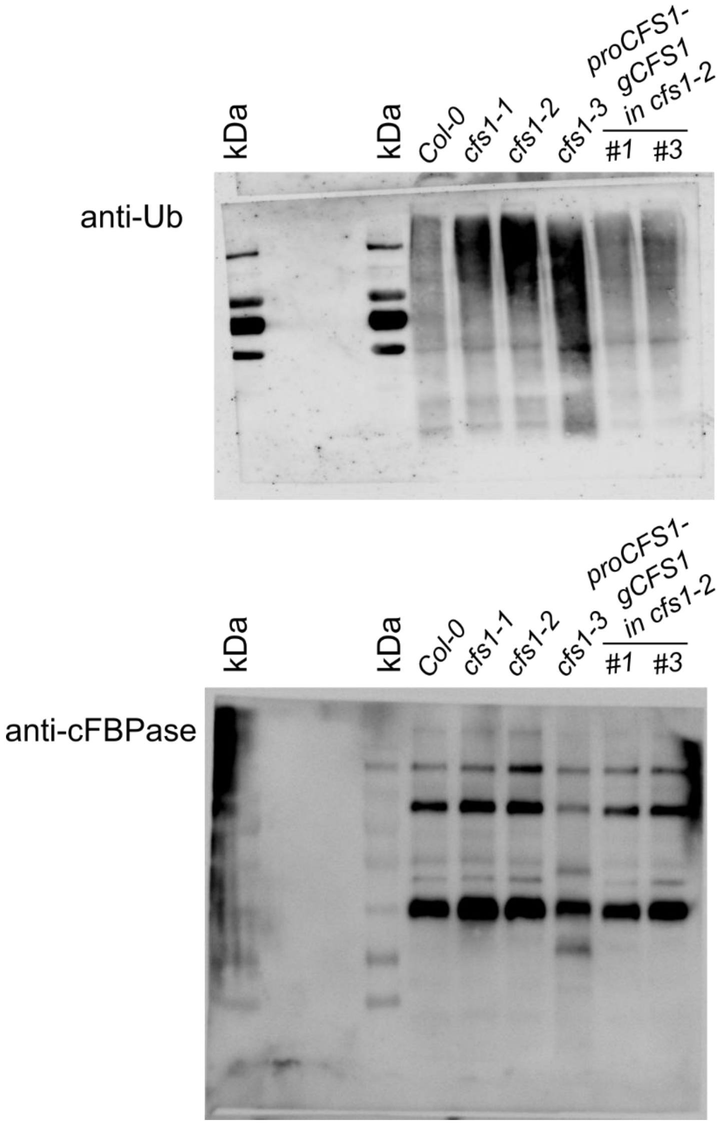

Supplementary figure S12: Immunoblot of anti-Ub reveals substantial accumulation of ubiquitinated proteins in leaf 1 and 2 of three-week old *cfs1* mutants compared to Col-0 and the complemented lines of the same stage. Anti-cFBPase is used as loading control.

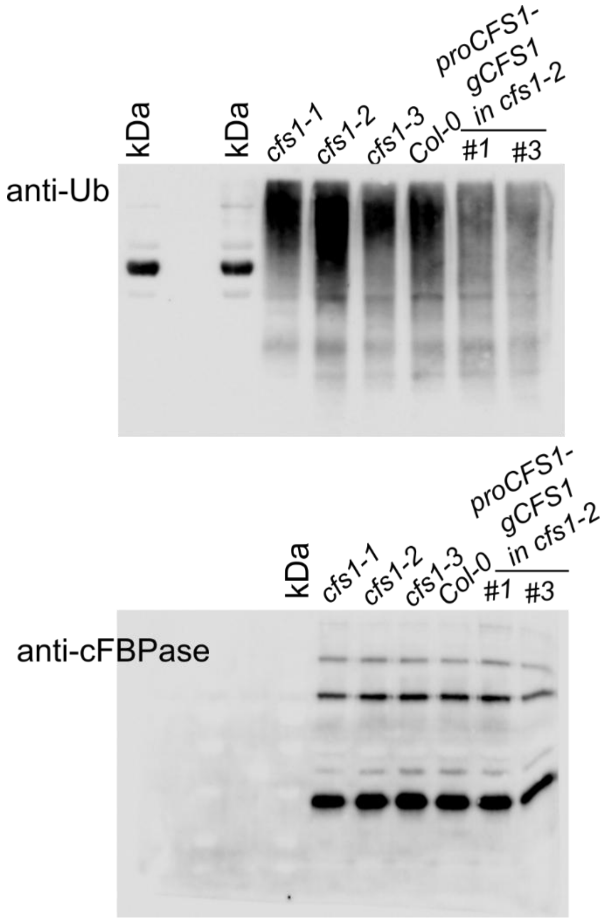

Supplementary figure S13: Immunoblot of anti-Ub reveals accumulation of ubiquitinated proteins in leaf 1 and 2 of two-week old *cfs1* mutants compared to Col-0 and the complemented lines of the same stage. Anti-cFBPase is used as loading control.

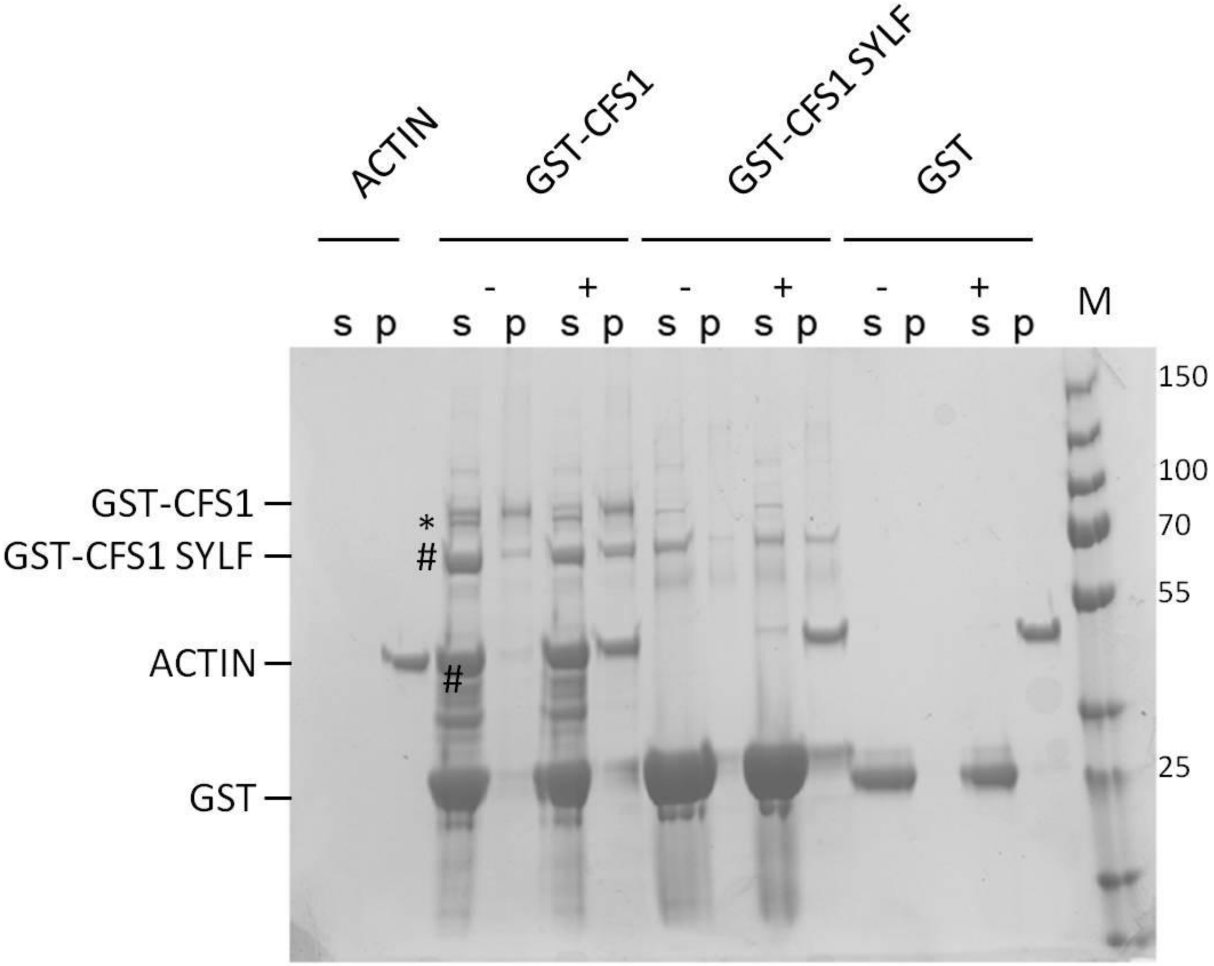

Supplementary figure S14: Coomassie stained gel of actin copreciptiation assays with GST-CFS1, GST-CFS1 SYLF and GST. Bands shown in figure 5 C and supplemental figure S5 are indicated on the left. (*= bacterial chaperone, #= degradation bands, p=pellet, s=supernatant, M=marker)

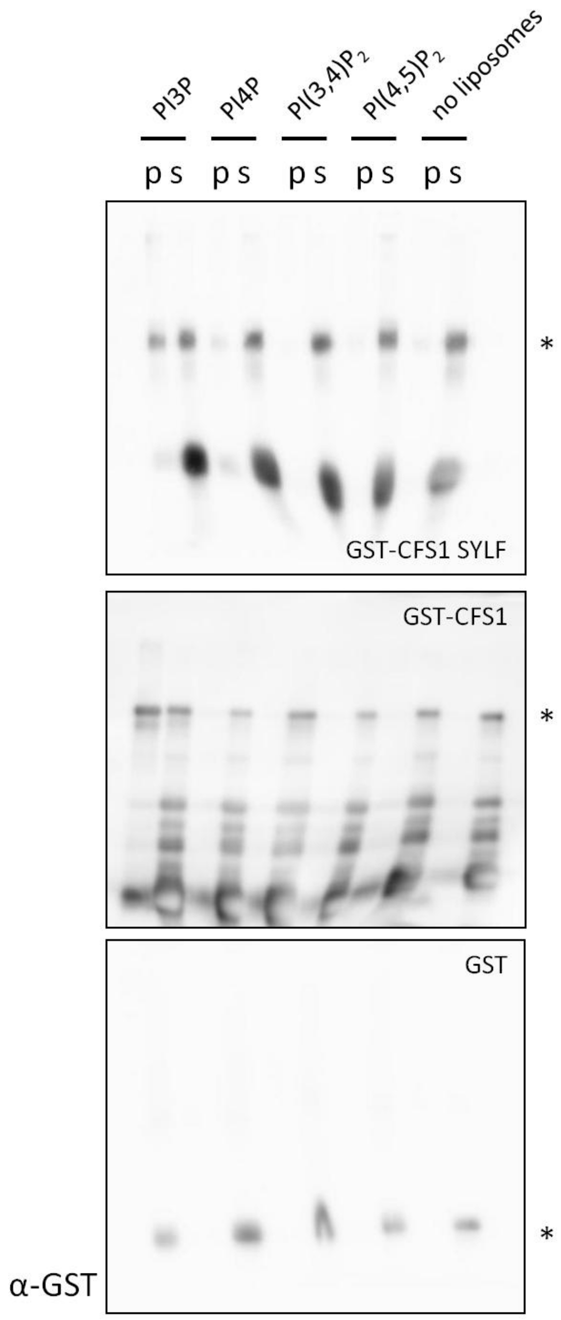

Supplementary figure S15: Immunoblot with anti-GST shows interactions of GST-CFS1 and GST-CFS1 SYLF with PI3P. Bands shown in figure 5 B are marked with asterisks. (p=pellet, s=supernatant).

Supplementary table 1: DNA primers used in this study

Name	AGI	Sequence (5'-3')	Reference
ACTIN semiRT fw	AT2G37620	TGCGACAATGGAACTGGAATG	
ACTIN semiRT re		GGATAGCATGTGGAAGTGCATAC	
AT1g29800_attB1	AT1G29800	GGGGACAAGTTTGTACAAAAAAGCAGGCTTA ATGGATGAAAGAGATCGAGA	
AT1g29800_attB2		GGGGACCACTTTGTACAAGAAAGCTGGGTT TTAGTCTTCAGACAATGGAG	
ATG8a qRT fw	AT4G21980	TAACCCTCTCGAGGCAAGG	Katsiarimpa et al., 2013
ATG8a qRT rv		TGTCAGGAACATCACTTTGTCC	
ATG8b qRT fw	AT4G04620	TTCAAGCTTTCTAATCCTCTGGA	
ATG8b qRT rv		TCCAGCTTTTTCCACAATCAC	
ATG8c qRT fw	AT1G62040	TTTCAAGTTGGAACACCCACTA	
ATG8c qRT rv		AGCTCTCTACGATCACTGGAA	
ATG8e qRT fw	AT2G45170	ACCCTGATCGAATTCCTGTG	
ATG8e qRT fw		TTAGGTCTGATGGCACAAGGT	
ATG8f qRT fw	AT4G16520	TCCTGATAGGATTCCGGTGA	
ATG8f qRT fw		AAACTGCCCCACAGTCAGAT	
ATG8g qRT fw	AT3G60640	CCCAAACATTGACAAGAAGAAA	
ATG8g qRT fw		CATACACAAACTGGCCTACCG	
ATG8h qRT fw	AT3G06420	GGGGATTGTTGTCAAGTCTTTC	
ATG8h qRT fw		TGCGTTTGAATATTTCTCAATGAT	
ATG8i qRT fw	AT3G15580	CGCTAAGTACCCTACTCGGATTC	
ATG8i qRT rv		TCTCTTGGAACCAGAAACTTCTTT	
eds1-2 fwd	AT3G48090	CCCTTTCTAGTTTCCTTGAGCTAAG	
eds1-2 rev		TCAGGTATCTGTTATTTCATCCATC	
eds5-1 fwd[1]	AT4G39030	CTGCACCTGTTTTTATCTCC	Nawrath et al., 2002
eds5-1 rev[1]		AATCTTCTCCACCGTGTATG	
EF1α f	AT5G60390	TGAGCACGCTCTTCTTGCTTTCA	Czechowski et al., 2005
EF1α r		GGTGGTGGCATCCATCTTGTTACA	
elch-m-a	AT3G12400	TCAAAAAAAGCACGTTCGGC	Spitzer et al.,

Name	AGI	Sequence (5'-3')	Reference
elch-m-s		ATGGTTCCCCCGCCGTCTAAT	2006
elch-wt-a		CGATTCTCAGGCCCTGATCC	
CFS1 3' fwd		GGCTCTGGTGTCTGCTATAC	
CFS1 3' rev		GTAATGTGAATGGAGATTACGG	
CFS1 5' fwd		TAAAATATTCCCCGAGGC	
CFS1 5' rev		CTACCACTACCATCATCA	
CFS1 aa238 +S GW RP	AT3G43230	GGGGACCACTTTGTACAAGAAAGCTGGGT TCTAAGGATCAAGCCTCTCGTAGC	
CFS1 aa238oS GW RP		GGGGACCACTTTGTACAAGAAAGCTGGGT TAGGATCAAGCCTCTCGTAGC	
CFS1 aa239 GW FP		GGGGACAAGTTTGTACAAAAAAGCAGGCTT AATGTTGCAATGTGTTCTTATTAA	
CFS1 GW FP		GGGGACAAGTTTGTACAAAAAAGCAGGCTT AATGGCTACTCTCAACGGAAA	
CFS1 GW oS RP		GGGGACCACTTTGTACAAGAAAGCTGGGT ACGGGCGCAAACGAGCATA	
Lbb1.3		ATTTTGCCGATTTCGGAAC	
NBR1 qPCRfwd	AT4G24690	GAGGAAATGGGTTTCAAGGA	
NBR1 qPCRrev		ATTGGATCCCACTCGCTAAC	
pCFS1_ApaI fwd		AAAGATAGACCGCATGAAAA	
pCFS1_ApaI rev		CGATTTATGGGATTGAAGAA	
pCFS1_BamHI fwd		CATAAGGAAAGTCCAAAGCA	
pCFS1_BamHI rev		AAGCAAAATCAAGATTTGAGGAT	
PR1 qRT fwd	AT2G14610	GATGTGCCAAAGTGAGGTGTAA	Katsiarimpa et al., 2013
PR1 qRT rev		TTCACATAATTCCCACGAGGA	
PR2 qRT fwd	AT3G57260	GTCTGAATCAAGGAGCTTAGCC	
PR2 qRT rev		AGCATACTCCGATTTGTCCAG	

Name	AGI	Sequence (5'-3')	Reference
PR5 qRT fwd	AT1G75040	GACTGTGGCGGTCTAAGATGT	
PR5 qRT rev		TGAATTCAGCCAGAGTGACG	
SAG13 qRT fwd	AT2G29350	AGGAAAACTCAACATCCTCGTC	
SAG13 qRT rev		GCTGACTCGAGATTTGTAGCC	
Salk_018265LP	AT3G43230	GAAGTTGAGATGGGGAAAACC	
Salk_018265RP		TTCGAATGTTTCGTTTCTTGG	
Salk_024058 RP	AT3G43230	CGGAAAAGCTTCTCCGTATTC	
Salk_024058LP		GCTCGACTAAGAACAGCATGC	
Salk_068647 LP	AT3G43230	AATCCCATAAATCGGATTTGC	
Salk_068647 RP		AACAGGGAGATTCAACCATCC	
sid2-1 fwd[3]	AT1G74710	TGCTTCTAGTGCTCGTGATA	
sid2-1 rev[3]		CACTCTGAAGATGGGTCACT	
WRKY53 qPCR fwd	AT4G23810	CGGAAGTCCGAGAAGTGAAG	
WRKY53 qPCR rev		TCTGACCACTTTGGTAACATCTTT	

[1]Amplicons from wild-type can be digested by LweI

[2] Dominant marker

[3] Amplicons from wild-type can be digested by MunI

Supplementary table 2: List of constructs and vectors used in this study

Gene	AGI	Vector	Vector source/ Construct reference
AT1G29800	AT1G29800	pAS, pACT	Clontec
ARA7	AT4G19640	pAMARENA	M Jakoby
ALIX	AT1G15130	pAS, pACT	Clontec/ Shahriari et al., 2011
SYP61	AT1G28490	pAMARENA	M Jakoby/ Herberth et al., 2012
ELC	AT3G12400	pAS, pACT	Clontec/ Shahriari et al., 2011
ELC		pEarleyGate104	Earley et al., 2006
ELC		pTREX-dest30-ntPrA, pcDNA3-Rluc-GW	Blasche and Koegl, 2013
ELC-LIKE	AT5G13860	pAS, pACT	Clontec/ Shahriari et al., 2011
ELC-LIKE		pTREX-dest30-ntPrA, pcDNA3-Rluc-GW	Blasche and Koegl, 2013
g-rb			Nelson et al., 2007
gCFS1	AT3G43230	pJET1.2	Thermo Scientific
CFS1		pAS, pACT	Clontec
		pENS-CFP	Feys et al., 2005
		pTREX-dest30-ntPrA, pcDNA3-Rluc-GW	Blasche and Koegl, 2013
CFS1 1-167		pENS-YFP	Feys et al., 2005
CFS1 1-167		pGEX2TMGW	Imre Sommsich and Bekir Ülker
CFS1 1-238		pENS-YFP	Feys et al., 2005
CFS1 1-238		pGEX2TMGW	Imre Sommsich and Bekir Ülker
CFS1 239-486		pENS-YFP	Feys et al., 2005
CFS1 239-486		pGEX2TMGW	Imre Sommsich and Bekir Ülker
Lifeact	Era et al., 2009	pBatTL -eGFP	Joachim Uhrig/ Steffens et al., 2014
LIP5	AT4G26750	pAS, pACT	Clontec
pCFS1		pJET1.2	Thermo Scientific
pCFS1		pAMPAT	GenBank: AY436765.1

Gene	AGI	Vector	Vector source/ Construct reference
pCFS1-gCFS1		pAMPAT	GenBank: AY436765.1
Vps20.1	AT5G63880	pAS, pACT	Clontec/ Shahriari et al., 2011
Vps20.2	AT5G09260	pAS, pACT	Clontec/ Shahriari et al., 2011
Vps2.1	AT2G06530	pAS, pACT	Clontec/ Shahriari et al., 2011
Vps22	AT4G27040	pAS, pACT	Clontec/ Shahriari et al., 2011
Vps2.2	AT5G44560	pAS, pACT	Clontec/ Shahriari et al., 2011
Vps2.3	AT1G03950	pAS, pACT	Clontec/ Shahriari et al., 2011
Vps28.1	AT4G21560	pAS, pACT	Clontec/ Shahriari et al., 2011
Vps28.2	AT4G05000	pAS, pACT	Clontec/ Shahriari et al., 2011
Vps32.1	AT2G19830	pAS, pACT	Clontec/ Shahriari et al., 2011
Vps36	AT5G04920	pAS, pACT	Clontec/ Shahriari et al., 2011
Vps37.1	AT3G53120	pAS, pACT	Clontec/ Shahriari et al., 2011
		pTREX-dest30-ntPrA, pcDNA3-Rluc-GW	Blasche and Koegl, 2013
Vps37.2	AT2G36680	pAS, pACT	Clontec/ Shahriari et al., 2011
Vps4	AT2G27600	pAS, pACT	Clontec/ Shahriari et al., 2011
Vps46.1	AT1G17730	pAS, pACT	Clontec/ Shahriari et al., 2011
Vps46.2	AT1G73030	pAS, pACT	Clontec/ Shahriari et al., 2011
Vps60.1	AT3G10640	pAS, pACT	Clontec/ Shahriari et al., 2011
Vps60.2	AT5G04850	pAS, pACT	Clontec/ Shahriari et al., 2011

Supplementary methods

Phylogenetic analysis, sequence alignments

Sequences of CFS1 and At1g29800 homologs from 44 taxa were retrieved by BLAST search (http://blast.ncbi.nlm.nih.gov/Blast.cgi?CMD=Web&PAGE_TYPE=BlastHome). Multiple alignments were created with the MAFFT tool (http://www.ebi.ac.uk/Tools/msa/mafft/) and manually refined in SeaView 4.5.4 (http://doua.prabi.fr/software/seaview). Gapped sites and poorly alignable regions were excluded from the analysis resulting in a 342 aligned amino acid positions.

The phylogenetic tree was created using a maximum likelihood (MLA) and a Bayesian (BA) analysis. ML was done with RAxML (raxmlGUI vs 1.3.1; https://sites.google.com/site/raxmlgui/) with 100 runs and 1000 bootstrap replicates ignoring bootstrap values below 50. BA was performed with two MCMC chains (500000 generations) with MrBayes 3.2.4 (http://mrbayes.sourceforge.net/) using the GTR+I+G model with WAG substitution frequencies. The first 200000 generations were discarded as 'burnin'. Posterior probabilities below 0.95 were ignored. Tree toplogy was built with RAxML.

Sequence alignments for the N-terminal comparisons of CFS1 homologs and At1g29800 were created with the ClustalW algorithm (Thompson et al., 1994) and visualized using BOXSHADE (http://www.ch.embnet.org/software/BOX_form.html).

Semi-quantitative RT-PCR

RNA was prepared as described in the main manuscript and 1µg was taken for cDNA synthesis. First strand cDNA was synthesised from total RNA by oligo-dT18 primers from RevertAid First Strand cDNA Synthesis Kit (Thermo Scientific) as described by the manufacturer. The reaction was performed at 42 °C for 60 min and terminated at 70°C for 5 min. PCR was performed for 20-35 cycles with primers for CFS1 that either span the T-DNA insertion sites or amplify the region 5’ or 3’ of the insertion. Aliquots were taken every two cycles. Reactions with *ACTIN* primers served as loading control. PCR products were visualized on 2% agarose gels by EtBr staining. All primers used are listed in Supplementary Table 1

Infection assays and ROS measurements

Spray infection assays were performed as in Zipfel et al. 2004 with the modifications described by Göhre et al., 2008. The experiment was performed in three biological replicates with 7-8 plants per genotype per replicate. The *Pseudomonas syringae* strain Pto DC3000 was sprayed onto the leaf surface of the plants at 108 cfu/ml and leaves harvested after 4 days. ROS burst

assays were performed as described in Frei dit Frey et al. 2012 with 6 biological replicates with 32 leaf discs stemming from at least eight plants per genotype and replicate.

Complexity measurements

Complexity measurements were performed by preparation of epidermal agarose imprints as described in Mathur and Koncz, 1997 and imaged with the DIC optics of a Leica DM5000B microscope. Circularity of the cells was determined using circularity function of the imageJ2 software.

Supplementary Literature

Blasche, S. and Koegl, M. (2013). Analysis of protein-protein interactions using LUMIER assays. Methods Mol. Biol. **1064**: 17–27.

Czechowski, T., Stitt, M., Altmann, T., Udvardi, M.K., and Scheible, W.-R. (2005). Genome-wide identification and testing of superior reference genes for transcript normalization in Arabidopsis. Plant Physiol. **139**: 5–17.

Earley, K.W., Haag, J.R., Pontes, O., Opper, K., Juehne, T., Song, K., and Pikaard, C.S. (2006). Gateway-compatible vectors for plant functional genomics and proteomics. Plant J. **45**: 616–629.

Era, A., Tominaga, M., Ebine, K., Awai, C., Saito, C., Ishizaki, K., Yamato, K.T., Kohchi, T., Nakano, A., and Ueda, T. (2009). Application of Lifeact reveals F-actin dynamics in Arabidopsis thaliana and the liverwort, Marchantia polymorpha. Plant Cell Physiol. **50**: 1041–8.

Feys, B.J., Wiermer, M., Bhat, R.A., Moisan, L.J., Medina-Escobar, N., Neu, C., Cabral, A., and Parker, J.E. (2005). Arabidopsis SENESCENCE-ASSOCIATED GENE101 stabilizes and signals within an ENHANCED DISEASE SUSCEPTIBILITY1 complex in plant innate immunity. Plant Cell **17**: 2601–13.

Fields, S. and Song, O. (1989). A novel genetic system to detect protein-protein interactions. Nature **340**: 245–6.

Göhre, V., Spallek, T., Häweker, H., Mersmann, S., Mentzel, T., Boller, T., de Torres, M., Mansfield, J.W., and Robatzek, S. (2008). Plant pattern-recognition receptor FLS2 is directed for degradation by the bacterial ubiquitin ligase AvrPtoB. Curr. Biol. **18**: 1824–32.

Herberth, S., Shahriari, M., Bruderek, M., Hessner, F., Müller, B., Hülskamp, M., and Schellmann, S. (2012). Artificial ubiquitylation is sufficient for sorting of a plasma membrane ATPase to the vacuolar lumen of Arabidopsis cells. Planta **236**: 63–77.

Katsiarimpa, A., Kalinowska, K., Anzenberger, F., Weis, C., Ostertag, M., Tsutsumi, C., Schwechheimer, C., Brunner, F., Hückelhoven, R., and Isono, E. (2013). The Deubiquitinating Enzyme AMSH1 and the ESCRT-III Subunit VPS2.1 Are Required for Autophagic Degradation in Arabidopsis. Plant Cell: 1–18.

Mathur, J. and Koncz, C. (1997). Method for preparation of epidermal imprints using agarose.

Biotechniques **22**: 280–282.

Nawrath, C., Heck, S., Parinthawong, N., and Métraux, J.-P. (2002). EDS5, an Essential Component of Salicylic Acid–Dependent Signaling for Disease Resistance in Arabidopsis, Is a Member of the MATE Transporter Family. Plant Cell **14**: 275–286.

Nelson, B.K., Cai, X., and Nebenführ, A. (2007). A multicolored set of in vivo organelle markers for co-localization studies in Arabidopsis and other plants. Plant J. **51**: 1126–36.

Pesch, M., Schultheiß, I., Digiuni, S., Uhrig, J.F., and Hülskamp, M. (2013). Mutual control of intracellular localisation of the patterning proteins AtMYC1, GL1 and TRY/CPC in Arabidopsis. Development **140**: 3456–67.

Shahriari, M., Richter, K., Keshavaiah, C., Sabovljevic, A., Huelskamp, M., and Schellmann, S. (2011). The Arabidopsis ESCRT protein-protein interaction network. Plant Mol. Biol. **76**: 85–96.

Spitzer, C., Schellmann, S., Sabovljevic, A., Shahriari, M., Keshavaiah, C., Bechtold, N., Herzog, M., Müller, S., Hanisch, F.-G., and Hülskamp, M. (2006). The Arabidopsis elch mutant reveals functions of an ESCRT component in cytokinesis. Development **133**: 4679–89.

Steffens, A., Jaegle, B., Tresch, A., Hülskamp, M., Jakoby, M., Hulskamp, M., and Jakoby, M. (2014). Processing-Body Movement in Arabidopsis Depends on an Interaction between Myosins and DECAPPING PROTEIN1. PLANT Physiol. **164**: 1879–1892.

Thompson, J.D., Higgins, D.G., and Gibson, T.J. (1994). CLUSTAL W: improving the sensitivity of progressive multiple sequence alignment through sequence weighting, position-specific gap penalties and weight matrix choice. Nucleic Acids Res. **22**: 4673–80.
